# Fortilin binds IRE1α and prevents ER stress from signaling apoptotic cell death

**DOI:** 10.1038/s41467-017-00029-1

**Published:** 2017-05-26

**Authors:** Decha Pinkaew, Abhijnan Chattopadhyay, Matthew D. King, Preedakorn Chunhacha, Zhihe Liu, Heather L. Stevenson, Yanjie Chen, Patuma Sinthujaroen, Owen M. McDougal, Ken Fujise

**Affiliations:** 1 Division of Cardiology, Department of Internal Medicine, University of Texas Medical Branch at Galveston, Galveston, Texas 77555 USA; 2 Department of Biochemistry and Molecular Biology, University of Texas Medical Branch at Galveston, Galveston, Texas 77555 USA; 3 Department of Chemistry and Biochemistry, Boise State University, Boise, Idaho 83725 USA; 4 Department of Pathology, University of Texas Medical Branch at Galveston, Galveston, Texas 77555 USA; 5 The Institute of Translational Sciences, University of Texas Medical Branch at Galveston, Galveston, Texas 77555 USA

## Abstract

The endoplasmic reticulum, the cytoplasmic organelle that matures a massive amount of nascent secretory polypeptides, is particularly sensitive to stress. Endoplasmic reticulum stress causes unfolded proteins to populate the organelle, eliciting the unfolded protein response. During the unfolded protein response, GRP78—an endoplasmic reticulum master stress regulator—detaches from three endoplasmic reticulum stress sensors (IRE1α, PERK, and ATF6) and allows them to activate the apoptotic signaling pathway. Fortilin, a pro-survival molecule, is known to inhibit apoptosis by binding and inhibiting p53, but its role in endoplasmic reticulum stress-induced apoptosis remains unknown. Here, we report that fortilin directly interacts with the cytoplasmic domain of IRE1α, inhibits both kinase and endoribonuclease (RNase) activities of the stress sensor, and protects cells against apoptotic cell death at both cellular and whole animal levels. Our data support a role of fortilin in the unfolded protein response and its potential participation in human diseases caused by unfolded protein response.

## Abstract (web summary)

IRE1α is an ER stress sensor, whose activity induces apoptosis. Here, the authors report that fortilin, a pro-survival factor, with yet unknown roles in ER stress, interacts with active IRE1α, inhibits both its kinase end RNase activities, and protects cells from apoptosis both in vitro and in vivo.

## Introduction

Precipitated by nutrient deprivation, hypoxia, and reactive oxygen species, endoplasmic reticulum (ER) stress causes protein folding to slow and unfolded proteins to accumulate in the organelle, eliciting the unfolded protein response (UPR). The UPR is a cellular process highly conserved across species that is designed to restore and enhance the ability of the ER to fold and process proteins and to avoid the catastrophic outcome (i.e., death of the organism) of uncontrolled and overwhelming accumulation of misfolded proteins^1^. During the UPR, GRP78 (also known as BiP)—an ER resident master stress regulator protein—detaches from three key ER transmembrane stress sensors (IRE1, PERK, and ATF6) to bind and sequester defective proteins. When freed from the binding and suppression of GRP78, IRE1, PERK, and ATF6 become activated and initiate the UPR^2^.

Mammalian IRE1 has two isoforms—widely expressed IRE1α^3^ and sparsely expressed IRE1β^4^. IRE1β is expressed only in the epithelium of the gastrointestinal tract^5^ and is absent in the liver and pancreas^5^. IRE1β processes 28S ribosomal RNA, but not X-box-binding protein 1 (XBP1) messenger RNA (mRNA)^6^, and participates in mucosal secretion^7^ and lipid transport in the gut^8^. On the other hand, IRE1α is ubiquitously expressed and plays a major role in how cells and organisms respond to ER stress^2^. The cytosolic portion of IRE1α contains the kinase and endoribonuclease (RNase) domains. After the luminal portion of IRE1α dissociates from GRP78, IRE1α oligomerizes and trans-autophosphorylates, leading to activation of its kinase and RNase domains. When activated, the RNase domain of IRE1α splices *XBP1* mRNA to produce *XBP1*s, which is an active transcriptional factor that induces genes related to ER membrane biogenesis and cell homeostasis^9^. The kinase domain of activated IRE1α (P-IRE1α), on the other hand, activates Jun N-terminal kinase (JNK) by recruiting first the scaffold protein TRAF2^10^ and then the apoptosis signal-regulating kinase (ASK1)^11^, which phosphorylates JNK and activates the JNK pro-apoptotic pathway^12^. Activated JNK not only phosphorylates and inactivates the anti-apoptotic proteins B-cell lymphoma (BCL)-2, BCL-XL, and myeloid cell leukemia protein 1 (MCL1), but it also phosphorylates and activates the BH-3-domain-only pro-apoptotic proteins such as BID and BIM^13^. The activation of IRE1α causes the cell to apoptose when pro-apoptotic predisposition overcomes homeostatic and reparatory propensity within the cell. However, the mechanism of this dichotomy is poorly understood^14^.

Originally cloned in 1989 as a molecule abundantly expressed in tumor cells^15^, fortilin is a 172-amino-acid (aa) polypeptide present in the nucleus, cytosol, and extracellular space^16, 17^. Fortilin is also known as translationally controlled tumor protein. While implicated in diverse cellular functions^18, 19^, fortilin possesses potent anti-apoptotic activity^16, 20–22^. Despite the well-documented anti-apoptotic activity of fortilin, its precise role in ER stress-induced apoptotic cell death remains unknown.

Herein we report that fortilin protects cells against ER stress-induced apoptosis by directly and preferentially binding P-IREα and preventing it from cleaving *XBP1* and activating the JNK apoptosis pathway. At the whole animal level, fortilin protected mice against liver failure and death induced by hepatocyte ER stress. We propose that the fortilin-IRE1α interaction is one of the important mechanisms by which cells mitigate ER stress-induced apoptotic cell death.

## Results

### ER stress translocates fortilin from nucleus to cytosol

**Fig. 1:**
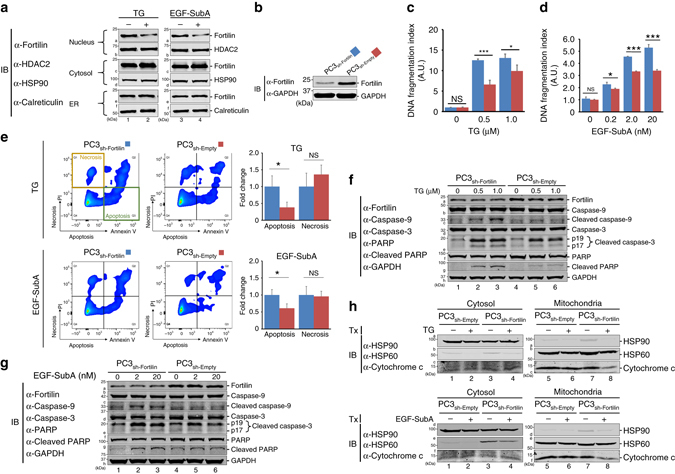
Fortilin protects cells against apoptosis under ER stress. **a** ER stress induced by thapsigargin (TG) and the epidermal growth factor (EGF) fused to the proteolytic A subunit of the bacterial AB_5_ toxin (SubA) (EGF-SubA) translocates fortilin from the nucleus to the cytosol. PC3 cells were challenged by either TG or EGF-SubA and their lysates were subjected to subcellular fractionation and immuno-blot analyses (IB). *HDAC2* histone deacetylase 2, *HSP90* heat shock protein 90. **b** Generation and characterization of fortilin-deficient PC3 cells. PC3 cells with decreased fortilin expression were generated by lentivirally introducing shRNA-fortilin into the cells and characterized by IB. **c**, **d** Fortilin protects cells against ER stress-induced apoptosis. PC3_sh-Fortilin_ and PC3_sh-Empty_ cells were challenged by either TG or EGF-SubA and subjected to the DNA fragmentation assay to evaluate the degree of apoptosis. *A.U.* arbitrary unit. Data were expressed as means ± s.d. (biological replicates [*n*] = 3) and analyzed by two-tailed unpaired *t*-test. *NS* not statistically significant; *P < 0.05; ***P < 0.005. **e** Fortilin protects cells against apoptosis, but not necrosis, under TG- and EGF-SubA-induced ER stress. PC3_sh-Fortilin_ and PC3_sh-Empty_ cells were challenged by either TG or EGF-SubA, stained with fluorescein isothiocyanate (FITC)-conjugated Annexin V and propidium iodide (PI), and analyzed by flow cytometry to evaluate the role of fortilin in the prevention of apoptosis and necrosis. Data were expressed as means ± s.d. (*n* = 3) and analyzed by two-tailed unpaired *t*-test. *NS* not statistically significant; *P < 0.05. **f**, **g** Fortilin protects cells against ER stress-induced cleavage of caspase-3, caspase-9, and PARP. PC3_sh-Fortilin_ and PC3_sh-Empty_ cells were challenged by either TG **f** or EGF-SubA **g** and subjected to IB to evaluate the status of the cleavage and activation of the caspases. The lack of fortilin predisposed PC3 cells to the cleavage of caspase-9, caspase-3, and poly ADP ribose polymerase (PARP) when challenged by TG- and EGF-SubA. *GAPDH* glyceraldehyde 3-phosphate dehydrogenase. **h** Fortilin protects cells against ER stress-induced release of cytochrome c from the mitochondria into the cytosol. PC3_sh-Fortilin_ and PC3_sh-Empty_ cells were challenged by either TG (the *top panel*) or EGF-SubA (the *bottom panel*). Cell lysates were fractionated into cytosolic and mitochondrial fractions. The levels of cytochrome c in the individual fractions were evaluated by IB. *Tx* treatment, *HSP60* heat shock protein 60

To test whether fortilin changes its intracellular localization upon ER stress, we stimulated the PC3 human prostate cancer cell line with either thapsigargin (TG) or the epidermal growth factor (EGF) fused to the proteolytic A subunit of a bacterial AB_5_ toxin (SubA) (EGF-SubA), subjected cells to subcellular fractionation, and quantified fortilin concentrations in the nuclear, cytosolic, and ER fractions using immunoblot analysis. TG is a well-characterized ER stress-inducing agent^23^ that induces ER stress in the cell by binding to and inhibiting Ca^2+^-ATPase, an ER resident transmembrane protein that maintains Ca^2+^ homeostasis^24^. EGF-SubA is an engineered fusion protein^25^. When exposed to EGF-SubA, cells expressing the EGF receptor internalize the fusion molecule into the cytosol. EGF-SubA is then retrogradely transported via the Golgi system to the ER lumen^26^, where it selectively and rapidly cleaves and destroys GRP78^25, 27^. Because GRP78 is the only known substrate of SubA^27^, EGF-SubA represents a highly specific inducer of ER stress. At the baseline, fortilin was present in all three fractions (Fig. 1a, a1, a3, c1, c3, e1, and e3; Supplementary Fig. 6). Upon ER stress induced by either TG or EGF-SubA, fortilin concentration decreased in the nuclear fractions (Fig. 1a, from a1 to a2; from a3 to a4) and increased in the cytosolic fractions (Fig. 1a, from c1 to c2; from c3 to c4). Consistently, immunocytochemistry of human osteosarcoma U2OS cells showed that TG-induced ER stress caused the fortilin signal in the nucleus to decrease and that in the perinuclear zone of the cytosol to increase (Supplementary Fig. 1A). Together, these data suggest that ER stress translocates fortilin from the nucleus to the ER region of the cytosol.

### Fortilin protects cells against ER-stress-induced apoptosis

To test whether fortilin can protect cells against ER stress-induced apoptosis, we generated PC3 cells lacking fortilin (PC3_sh-Fortilin_) by lentivirally introducing short-hairpin-RNA against fortilin (sh-Fortilin) into the cell. PC3_sh-Fortilin_ expressed significantly less fortilin than did the control cells (PC_sh-Empty_) (Fig. 1b; Supplementary Fig. 6). Next, we challenged these cells with either TG (Fig. 1c) or EGF-SubA (Fig. 1d) and quantified the degree of DNA fragmentation. Both TG and EGF-SubA dose-dependently induced DNA fragmentation in both PC3_sh-Empty_ and PC3_sh-Fortilin_ cells (Fig. 1c, d). However, PC3_sh-Empty_ cells exhibited significantly less DNA fragmentation than did PC3_sh-Fortilin_ cells at all concentrations of TG and EGF-SubA (Fig. 1c, d). These data indicate that fortilin protected PC3 cells against ER stress-induced apoptosis. Next, we generated U2OS cells overexpressing fortilin (U2OS_Fortilin-HA_) and control cells (U2OS_Empty-HA_) (Supplementary Figs. 1B and 9), challenged the cells with TG, and assessed the survival (Supplementary Fig. 1C) and degree of DNA fragmentation (Supplementary Fig. 1D) of these cells. Fortilin protected the U2OS cells against TG-induced cell death and DNA fragmentation (Supplementary Fig. 1C, D).

To evaluate how fortilin modulates ER stress-induced apoptosis and necrosis, we challenged PC3_sh-Fortilin_ and PC3_sh-Empty_ cells with either TG or EGF-SubA, stained them with fluorescein isothiocyanate (FITC)-conjugated Annexin V and propidium iodide (PI), and analyzed them using flow cytometry. Upon TG and EGF-SubA challenge, PC3_sh-Fortilin_ cells bound more Annexin V than did PC3_sh-Empty_ cells (Fig. 1e, Apoptosis; *P < 0.05 by two-tailed unpaired *t*-test), although there was no difference in PI levels between the two groups of cells (Fig. 1e, Necrosis; NS = not statistically significant). These data suggest that fortilin protected PC3 cells against ER stress-induced apoptosis but not against necrosis (Fig. 1e).

To assess the role of fortilin in the ER stress-induced activation of caspases, we challenged PC3_sh-Fortilin_ and PC3_sh-Empty_ cells with either TG or EGF-SubA, and subjected the cell lysates to immunoblot analysis (Fig. 1f, g; Supplementary Fig. 6). Both TG and EGF-SubA induced more cleavage of caspases-9 and -3 and PARP in PC3_sh-Fortilin_ than in PC3_sh-Empty_ cells (Fig. 1f for TG: caspase-9, c2 and c3 vs. c5 and c6; caspase-3, e2 and e3 vs. e5 and e6; PARP, g2 and g3 vs. g5 and g6, respectively) (Fig. 1g for EGF-SubA: caspase-9, c2 and c3 vs. c5 and c6; caspase-3, e2 and e3 vs. e5 and e6; PARP, g2 and g3 vs. g5 and g6, respectively). Next, we challenged U2OS_Empty-HA_ and U2OS_Fortilin-HA_ cells with TG and quantified the activities of caspase-3, -8, and -9. TG dose-dependently activated caspase-9 and -3 but not -8, as previously reported^28^ (Supplementary Fig. 1E). In this system, fortilin protected TG-challenged U2OS cells against the activation of caspases-9 and -3 (Supplementary Fig. 1E, ***P < 0.005; *P < 0.05, both by two-tailed unpaired *t*-test).

Next, we challenged PC3_sh-Fortilin_ and PC3_sh-Empty_ cells with either TG or EGF-SubA, fractionated the lysates into cytosolic and mitochondrial fractions, and subjected them to immunoblot analysis using anti-cytochrome c antibody. The cytosolic fraction of PC3_sh-Fortilin_ cells contained a greater amount of cytochrome c upon TG/EGF-SubA simulation than the control (Fig. 1h; c3 vs. c4 for TG; f3 vs. f4 for EGF-SubA; Supplementary Fig. 7). The mitochondrial fraction of PC3_sh-Fortilin_ cells contained a lesser amount of cytochrome c upon TG/EGF-SubA simulation than the baseline (Fig. 1h; c7 vs. c8 for TG; f7 vs. f8 for EGF-SubA). TG/EGF-SubA stimulation did not change the amount of cytochrome c in the cytosol or mitochondria in PC3_sh-Empty_ cells (Fig. 1h; c1 vs. c2, c5 vs. c6 for TG; f1 vs. f2, f5 vs. f6 for EGF-SubA).These data indicate that fortilin prevents cytochrome c release from mitochondria to the cytosol in TG/EGF-SubA-stimulated PC3 cells.

### Fortilin prevents ER stress from activating IRE1α

**Fig. 2:**
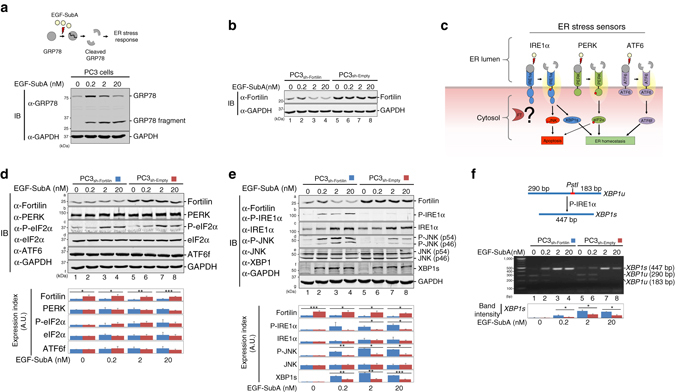
Fortilin selectively inhibits IRE1α signaling to protect against ER stress-induced apoptosis. **a** EGF-SubA dose-dependently cleaves GRP78 in PC3 cells. Wild-type PC3 cells were treated with the indicated concentrations of EGF-SubA, and their lysates were subjected to IB. **b** EGF-SubA treatment does not change the expression levels of fortilin in PC3 cells. PC3_sh-Empty_ and PC3_sh-Fortilin_ cells were treated with the indicated concentrations of EGF-SubA for 24 h and subjected to IB. **c** Three branches of ER stress signaling pathways. IRE1α, PERK, and ATF6 are the ER resident transmembrane proteins that signal ER stress when activated. IRE1α and PERK, but not ATF6, are known to induce apoptosis. **d** Fortilin does not regulate the PERK and ATF6 signaling pathways. PC3_sh-Empty_ and PC3_sh-Fortilin_ cells were treated with the indicated concentrations of EGF-SubA, and their lysates were subjected to quantitative IB for PERK, its downstream molecule eIF2α, and fragmented and activated ATF6 (ATF6f). Data were expressed as means ± s.d. (*n* = 3) and analyzed by two-tailed unpaired *t*-test. *P < 0.05; **P < 0.01; ***P < 0.005. **e** Fortilin inhibits the IRE1α ER stress signaling pathway. PC3_sh-Empty_ and PC3_sh-Fortilin_ cells were treated with the indicated concentrations of EGF-SubA and subjected to IB for IRE1α and its downstream molecules JNK and spliced XBP1 (XBP1s). P-IRE1α is the activated form of IRE1α, and P-JNK is the activated form of JNK. Data were expressed as means ± s.d. (*n* = 3, except for P-IRE1α and XBP1s where *n* = 2) and analyzed by two-tailed unpaired *t*-test. *P < 0.05; **P < 0.01; ***P < 0.005. **f** Fortilin inhibits the processing by IRE1α of *XBP1* mRNA. PC3_sh-Empty_ and PC3_sh-Fortilin_ cells were treated with the indicated concentrations of EGF-SubA. The cDNA from total RNA from these cells was subjected to PCR and *Pst1* restriction digestion (CTGCA|G) to semi-quantitatively evaluate the levels of *XBP1s*. Data were expressed as means ± s.d. (*n* = 2) and analyzed by two-tailed unpaired *t*-test. *P < 0.05. *XBP1u* unspliced *XBP1*

EGF-SubA targets a single protein, namely GRP78, in mammalian cells^27^. Cells expressing a SubA-resistant mutant GRP78 (GRP78^L416D^) are immune to EGF-SubA^27^, suggesting that EGF-SubA is a selective ER stress-inducing agent with minimum off-target effects. To further study the role of fortilin in ER stress signaling in a system free of potential downstream effects of TG-induced perturbances in Ca^2+^ ^24^, we performed all remaining experiments using EGF-SubA. To confirm that EGF-SubA cleaves GRP78 in PC3 cells, we incubated PC3 cells with 0–20 nM of EGF-SubA and subjected the lysates to immunoblot analysis using anti-GRP78 antibody. EGF-SubA dose-dependently cleaved GRP78 in PC3 cells (Fig. 2a; Supplementary Fig. 7), but treatment with EGF-SubA did not affect the expression levels of fortilin in either PC3_sh-Fortilin_ or PC3_sh-Empty_ cells at 24 h (Fig. 2b, Fortilin in lanes 1–4 and lanes 5–8, respectively; Supplementary Fig. 7).

During the UPR, GRP78 detaches from the ER transmembrane stress sensors IRE1α, PERK, and ATF6 to bind and sequester defective proteins in the ER lumen. When IRE1α, PERK, and ATF6 are freed from the binding and suppression of GRP78, they activate various ER stress pathways^2^. It has been shown that IRE1α^12^ and PERK^29^, but not ATF6^30^, signal ER stress-induced apoptosis (Fig. 2c). Because fortilin protects cells against ER stress-induced apoptosis (Fig. 1c–h, Supplementary Fig. 1D, E), we first assessed whether fortilin blocks either the PERK or IRE1α pathway by treating PC3_sh-Fortilin_ and PC3_sh-Empty_ cells with EGF-SubA and subjecting them to immunoblot analysis (Fig. 2d, e; Supplementary Fig. 7). Both PC3_sh-Fortilin_ and PC3_sh-Empty_ cells expressed similar amounts of PERK and eukaryotic translation initiation factor alpha (eIF2α), regardless of the concentrations of EGF-SubA (Fig. 2d, rows b and d, and the bottom graph). The cellular levels of phosphorylated eIF2α (P-eIF2α), the active form of eIF2α, dose-dependently increased in response to EGF-SubA, and there was no detectable difference in P-eIF2α levels between PC3_sh-Fortilin_ and PC3_sh-Empty_ cells (Fig. 2d, row c of lanes 1–4 vs. lanes 5–8, and the bottom graph). Both PC3_sh-Fortilin_ and PC3_sh-Empty_ cells expressed similar amounts of IRE1α and JNK (both p54 and p46), regardless of the concentrations of EGF-SubA (Fig. 2e, rows c and e of lanes 1–4 vs. lanes 5–8, and the bottom graph). Strikingly, however, PC3_sh-Fortilin_ cells expressed greater amounts of phosphorylated and activated forms of IRE1α (P-IRE1α) and JNK (P-JNK) than did PC3_sh-Empty_ cells (Fig. 2e, rows b and d of lanes 2–4 vs. lanes 6–8). Enzyme-linked immunosorbent assay (ELISA) analysis of the cell lysates from PC3_sh-Fortilin_ and PC3_sh-Empty_ cells treated with various concentrations of EGF-SubA also showed that fortilin protected PC3 cells against the phosphorylation of JNK (Supplementary Fig. 2). Furthermore, the protein expression and mRNA levels of XBP1s—the active form of XBP1 generated by P-IRE1α—were greater in PC3_sh-Fortilin_ than in PC3_sh-Empty_ cells (Fig. 2e, row f of lanes 2–4 vs. lanes 6–8 and Fig. 2f, lanes 2–4 vs. lanes 6–8, respectively, and the bottom graphs). Consistent with previous reports that ATF6 does not participate in ER stress-induced apoptosis^30^, we found that fortilin did not affect the cellular levels of active and fragmented ATF6 (ATF6f) (Fig. 2d, row e of lanes 1–4 and lanes 5–8). These data suggest that fortilin inhibits the IRE1α branch, but not the PERK or ATF6 branch, of the ER stress signaling pathway and that the anti-apoptotic activities (Fig. 1c–h, Supplementary Fig. 1D, E) of fortilin in the setting of ER stress are mediated through its inhibition of the IRE1α signaling pathway.

### Fortilin binds the cytosolic domain of IRE1α

**Fig. 3:**
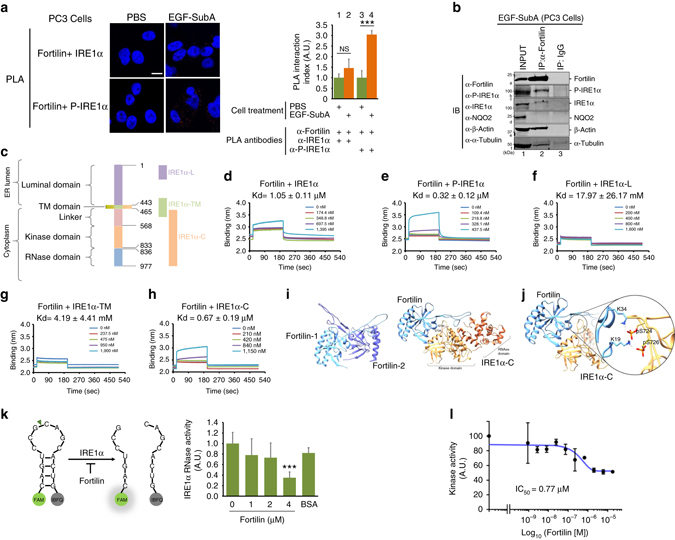
Fortilin interacts with the cytosolic domain of IRE1α and inhibits its protein kinase and RNase activities. **a** Proximity ligation assay (PLA) shows a specific interaction between fortilin and P-IRE1α in EGF-SubA-treated PC3 cells. The cells were treated with 2 nM EGF-SubA for 24 h and subjected to PLA, using anti-IREα and anti-P-IRE1α antibodies to evaluate fortilin-IRE1α and fortilin-P-IRE1α interaction, respectively. PLA interaction indices were calculated by dividing the number of red dots by the number of nuclei, expressed as means ± s.d. (*n* = 3), and analyzed by two-tailed unpaired *t*-test. *NS* not statistically significant; ***P < 0.005. Scale bar = 10 µm. **b** Fortilin co-immunoprecipitates P-IRE1α. PC3 cells were treated with 2 nM EGF-SubA for 24 h, lysed and subjected to immunoprecipitation (IP). **c** Domain structure of human IRE1α. Human IRE1α consists of the ER luminal domain (aa 1–443), transmembrane domain (aa 444–464), linker region (aa 465–567), kinase domain (aa 568–833), and endoribonuclease (RNase) domain (aa 836–997). The following recombinant proteins were used for biolayer interferometry: full-length IRE1α (aa 1-977), IRE1α-Myc-DDK (aa 1–977); IRE1α-L, GST-IRE1α (aa 1–70); IRE1α-TM, GST-IRE1α (aa 401–500); and IRE1α-C, GST-IRE1α (aa 468–977). **d**–**h** Fortilin binds to P-IRE1α through its cytosolic domain. Biotinylated fortilin was immobilized to the streptavidin biosensor. Recombinant IRE1α, either full-length or fragment, was applied to the biosensor at various concentrations, and dissociation constants (Kds, expressed as mean ± s.d., *n* = 3) were derived. **i** Lowest energy binding pose of fortilin (*blue*) with cytosolic domain of IRE1α (*green*) (the *right panel*) presented with that of a fortilin-fortilin dimer (the *left panel*). **j** Intermolecular interactions between phosphorylated serine724 (pS^724^) and serine726 (pS^726^) of the cytosolic domain of IRE1α with lysine residues (K^19^ and K^34^) of fortilin. **k** Fortilin inhibits the RNase activity of IRE1α. An in vitro IRE1α RNase activity assay was performed by incubating IRE1α with human recombinant fortilin and the substrate fluorescently tagged *XBP1* RNA stem loop, the cleavage of which would allow the fluorescein amidite (FAM) to fluoresce. Data were expressed as means ± s.d. (*n* = 4) and analyzed by two-tailed unpaired *t*-test. ***P < 0.005. **l** Fortilin inhibits the kinase activity of IRE1α. An in vitro IRE1α kinase activity assay was performed by incubating IRE1α with [γ-^33^P]ATP, recombinant fortilin, and myelin basic protein (MBP) as a substrate of the kinase in the kinase reaction buffer. The phosphorylation index was calculated by dividing the radioactivity of MBP for a given fortilin concentration by that of the vehicle control and expressed as means ± s.d. (*n* = 2) from which half maximal inhibitory concentration (IC_50_) was calculated

To investigate how fortilin selectively and negatively regulates IRE1α and its signaling pathway, we tested whether fortilin binds IRE1α using the in situ proximity ligation assay (PLA)^31^. We seeded wild-type PC3 cells onto glass slides and treated them with either phosphate buffered saline (PBS) or EGF-SubA before subjecting them to the PLA using (a) mouse anti-fortilin and (b) rabbit anti-IRE1α or anti-P-IRE1α antibodies (where anti-IRE1α detects total IRE1α—both phosphorylated and unphosphorylated—and anti-P-IRE1α detects only P-IRE1α). Each red dot in Fig. 3a indicates that a fortilin molecule was within about 30 nm of an IRE1α molecule. We counted the number of red dots and divided it by the number of nuclei to calculate the PLA interaction index. EGF-SubA-induced ER stress drastically increased the number of interactions between fortilin and P-IRE1α (Fig. 3a, right panel, bars 3 vs. 4) but not the number of interactions between fortilin and IRE1α (Fig. 3a, right panel, bars 1 vs. 2).

To further evaluate the interaction between fortilin and P-IRE1α in vivo, we challenged PC3 cells with 2 nM EGF-SubA for 24 h and incubated the cleared total cell lysates with either rabbit anti-fortilin monoclonal antibody (Clone EPR5540) or rabbit IgG. We precipitated the formed protein–antibody complexes with Dynabeads™ Protein G (Dynal-ThermoFisher Scientific, Waltham, MA, USA), washed them extensively, eluted them into the sodium dodecyl sulfate (SDS) loading buffer, and subjected them to SDS-polyacrylamide gel electrophoresis (PAGE) and immunoblotting. The rabbit anti-fortilin antibody, but not rabbit IgG, successfully immunoprecipitated fortilin (Fig. 3b, band a2; Supplementary Fig. 7). Both β-actin and α-tubulin—known fortilin binding proteins^32,  33^—were co-immunoprecipitated with fortilin (Fig. 3b, band e2 and f2). NQO2—a protein known not to interact with fortilin^34^—was not co-immunoprecipitated, suggesting that the wash condition was sufficiently stringent (Fig. 3b, no band in d2). In this system, both anti-P-IRE1α and anti-IRE1α antibodies detected discrete bands at the expected molecular weights (Fig. 3b, bands b2 and c2, respectively). The immunoblot signals of fortilin, P-IRE1α, IRE1α, β-actin, and α-tubulin (Fig. 3b, bands a2, b2, c2, e2, and f2, respectively) were from specific protein–protein interactions and did not represent non-specific interaction between the proteins and the beads because there were no immuneblot signals in the sample incubated with the same beads and IgG (Fig. 3b, a3, b3, c3, d3, e3, and f3). Because anti-IRE1α antibody is known to detect both non-phosphorylated and phosphorylated IRE1α, band c2 (Fig. 3b) could be either non-phosphorylated or phosphorylated IRE1α. Importantly, however, the presence of a band in the membrane probed by anti-P-IRE1α (Fig. 3b, band b2) shows the presence of a specific interaction between fortilin and P-IRE1α. We found the same in PC3 cells treated by TG (Supplementary Figs. 3A and 9). These data, when taken together, suggest that fortilin specifically interacts with P-IRE1α.

IRE1α is a transmembrane protein consisting of five distinct domains—luminal, transmembrane, linker, kinase, and RNase domains^35^ (Fig. 3c). To further characterize the direct physical interaction between fortilin and P-IRE1α, we tested if biolayer interferometry (BLItz, ForteBio, Menlo Park, CA, USA)^36^ could be used to detect the interaction between fortilin and its binding partners. We first biotinylated and immobilized recombinant human fortilin (Supplementary Fig. 3B, lane 9) to the streptavidin-coated biosensor. BLItz appropriately detected the interaction between fortilin on the biosensor and anti-fortilin monoclonal antibody in the aqueous phase at the dissociation constant (Kd) of 71.46 ± 0.04 nM (mean values ± s.d., *n* = 3, Supplementary Fig. 3C), whereas no meaningful interaction between fortilin and albumin was detected (Kd = 1.05 ± 0.11 mM, mean values ± s.d. *n* = 3, Supplementary Fig. 3D). Next, we subjected human recombinant fortilin and IRE1α and its deletion mutants to the same biolayer interferometry (BLItz)^36^. Five different recombinant preparations of IRE1α were tested: IRE1α (dephosphorylated IRE1α, 1–977 aa, see Supplementary Figs. 3E and 9), P-IRE1α (phosphorylated IRE1α, 1–977 aa), IRE1α-L (the N-terminal, luminal portion of IRE1α, 1–70 aa), IRE1α-TM (the transmembrane portion of IRE1α, 401–500 aa), and IRE1α-C (the cytosolic portion of IRE1α, 468–977 aa)(Fig. 3c and Supplementary Fig. 3B, lanes 2, 5, 6, and 7). Fortilin bound only weakly to IRE1α (Fig. 3d) but tightly to P-IRE1α (Fig. 3e). Further, fortilin did not bind the luminal or transmembrane portions of IRE1α (Fig. 3f, g) but bound the cytosolic portion of IRE1α (Fig. 3h). These data suggest that fortilin specifically and directly binds to the activated and phosphorylated form of IRE1α (P-IRE1α) through its cytosolic domain (Fig. 3c–h and Supplementary Fig. 3G).

To further verify the binding of fortilin to IRE1α, we performed molecular docking and molecular dynamics (MD) experiments using the methods described in detail in the Methods section. Because fortilin has been shown to dimerize^37^, we performed a molecular docking study for both dimerized fortilins (fortilin:fortilin) and the fortilin–IRE1α-C complex (fortilin:IRE1α-C). The binding surface of fortilin that is used to interact with another fortilin for dimerization (Fig. 3i, the left panel) was nearly identical to that used to interact with IRE1α-C (Fig. 3i, the right panel). The relative binding favorability scores for the fortilin:fortilin and fortilin:IRE1α-C complexes were calculated to be −879.1 and −945.3 kcal mol^−1^, respectively, suggesting that the fortilin:IRE1α-C complex was more thermodynamically stable than the fortilin:fortilin complex. We then inspected the docking pose of the fortilin:IRE1α-C complex and found that the main phosphorylation sites (S^724^ and S^726^) of IRE1α^38^ were positioned in close proximity to the binding surface of fortilin (especially K^19^ and K^34^) regardless of their phosphorylation status, suggesting that the binding of fortilin to IRE1α blocks the access of anther IRE1α to the phosphorylation sites (S^724^ and S^726^) of the molecule for trans-autophophorylation. The adenosine diphosphate (ADP) binding region of the kinase domain (R^600^, E^612^, D^688^, N^693^, and D^711^)^38^ and the RNA processing region of the RNase domain (Y^892^, R^904^, N^905^, and H^909^)^38^ were not positioned near the binding surface of fortilin, although it is likely that binding of fortilin to the key phosphorylation sites of IRE1α induces conformational changes to disrupt the functionality of these regions^38^.

Next, we compared the thermodynamic stability of the fortilin–P-IRE1α complex with that of the fortilin–IRE1α complex using MD forward and reverse binding free energy simulations. We found that the fortilin-P–IRE1α complex was substantially more thermodynamically stable than the fortilin–IRE1α complex by a Δ*G*_bind_ value of −15.48 kcal mol^−1^ (Supplementary Table 1). The free energy change (Δ*G*_2_) due to phosphorylation of IRE1α in the bound state with fortilin was −602.61 ± 1.02 kcal mol^−1^ (mean values ± s.e.), whereas the free energy change (Δ*G*_1_) due to phosphorylation of unbound IRE1α was −587.13 ± 1.01 kcal mol^−1^ (mean values ± s.e.). Both the forward and reverse simulations, which correspond to phosphorylation and dephosphorylation of S^724^ and S^726^ of IRE1α, resulted in comparable values, thus supporting the validity of the calculations. The higher thermodynamic stability of the fortilin–P-IRE1α complex compared to that of fortilin–IRE1α may be due to the strong electrostatic forces between the positively charged lysine residues (K^19^ and K^34^) of fortilin and the negative charges of the phosphoserine residues (S^724^ and S^726^) (Fig. 3j). These MD data suggest that fortilin binds IRE1α in a thermodynamically stable fashion, that fortilin binds preferentially to P-IRE1α over IRE1α, and that fortilin binding to P-IRE1α occludes the key phosphorylation sites (S^724^ and S^726^) of the molecule and thus can prevent IRE1α dimerization and subsequent trans-autophosphorylation.

### Fortilin inhibits both kinase and RNase activities of IRE1α

The cytosolic domain of IRE1α contains the kinase and RNase domains (Fig. 3c). Because fortilin prevented EGF-SubA from phosphorylating IRE1α (row b of Fig. 2e and Supplementary Fig. 2) and splicing *XBP1* to form *XBP1s* (Fig. 2f and row f of Fig. 2e), we tested whether fortilin inhibits the kinase and RNase activities of IRE1α through its direct interaction with the ER stress sensor molecule. We first assessed the RNase activity of IRE1α in the presence of fortilin in vitro using a synthetic mRNA stem loop corresponding to the *XBP1* substrate sequence^39^. The stem loop incorporates a fluorescein amidite fluorophore (FAM) on its 5′ end and the Iowa Black FQ (IBFQ) quencher on its 3′ end, resulting in green fluorescence upon site-specific cleavage by IRE1α (Fig. 3k, left panel). In this system, fortilin significantly inhibited the RNase activity of IRE1α (Fig. 3k, right panel, no fortilin vs. 4 µM of fortilin; ***P < 0.005 by two-tailed unpaired *t*-test). Next, we evaluated the effect of recombinant fortilin on the ability of IRE1α to phosphorylate myelin basic protein (MBP) in vitro. MBP was used as a generic substrate of serine/threonine kinases such as IRE1α^40, 41^ because there is no specific substrate identified for IRE1α, other than IRE1α itself^42^. We performed a standard kinase assay using [γ-^33^P]ATP as previously described^41^ and found that fortilin dose-dependently prevented IRE1α from incorporating γ-^33^P into MBP, with the half maximal inhibitory concentration (IC_50_) being 0.77 µM (Fig. 3l). We also assessed the phosphorylation of MBP by IRE1α by immunoblot analysis using anti-phosphoserine antibody (Supplementary Figs. 3F and 9). At baseline, mild phosphorylation was noted in MBP (Supplementary Fig. 3F, P-MBP, lane 2). P-IRE1α increased the phosphorylation of MBP (Supplementary Fig. 3F, P-MBP, lane 4 vs. lane 2). In this system, fortilin decreased the phosphorylation of MBP (Supplementary Fig. 3F, P-MBP, lanes 4–8). These data suggest that fortilin binds and inhibits both the kinase and RNase activities of IRE1α.

### Fortilin saves mice from ER stress-induced liver failure

**Fig. 4:**
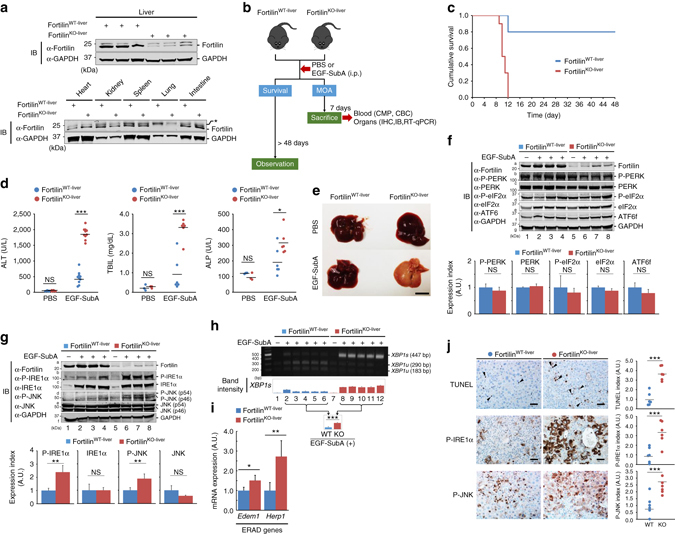
Fortilin protects the whole animal against ER stress-induced liver failure and death by negatively regulating the IRE1α stress sensor pathway. **a** Liver-specific absence of fortilin in fortilin^KO-liver^ mice. *, non-specific bands. **b** Experimental protocol. *i.p.* intraperitoneally, *MOA* mechanism of action, *CMP* complete metabolic panel, *CBC* complete blood count, *IHC* immunohistochemistry. **c** Higher survival rates of fortilin^WT-liver^ than fortilin^KO-liver^ mice after EGF-SubA challenge. A Kaplan–Meier survival curve and log-rank test showed the significantly better survival of fortilin^WT-liver^ mice than that of fortilin^KO-liver^ mice (*n* = 10, *P* < 0.001). **d** Massive liver damage of EGF-SubA-treated fortilin^KO-liver^ mice. The sera from fortilin^WT-liver^ and fortilin^KO-liver^ mice, treated with either PBS or EGF-SubA, were assayed for alanine aminotransferase (ALT; *n* = 5 and 10, for PBS and EGF-SubA, respectively), total bilirubin (TBIL; *n* = 3 and 6), and alkaline phosphatase (ALP; *n* = 3 and 6). Data were expressed as mean and analyzed by two-tailed unpaired *t*-test. *NS* not statistically significant; *P < 0.05; ***P < 0.005. **e** Drastic gross pathological change in the liver of EGF-SubA-treated fortilin^KO-liver^ mice. Scale bar = 10 mm. **f** Fortilin fails to negatively regulate the PERK and ATF6 signaling pathways in the EGF-SubA-challenged liver. The total lysates from the livers of fortilin^WT-liver^ and fortilin^KO-liver^ mice, after the indicated treatments, were subjected to IB. Data were expressed as means ± s.d. (*n* = 3) and analyzed by two-tailed unpaired *t*-test. *NS* not statistically significant. **g** Fortilin inhibits the IRE1α signaling pathway in the EGF-SubA-challenged liver. Data were expressed as means ± s.d. (*n* = 3) and analyzed by two-tailed unpaired *t*-test. *NS* not statistically significant; **P < 0.01. **h** Fortilin blocks IRE1α-mediated splicing of *XBP1* mRNA in the liver of mice under ER stress. The amounts of *XBP1s* and *XBP1u* were quantified by quantitative densitometry. Data were expressed as means ± s.d. (*n* = 5) and analyzed by two-tailed unpaired *t*-test. ***P < 0.005. **i** Fortilin inhibits the expression of XBP1s-inducible genes in the liver of mice under ER stress. Expression levels of ER-associated degradation (ERAD) genes—*Edem1* and *Herp1*—in the liver of EGF-SubA-challenged fortilin^WT-liver^ and fortilin^KO-liver^ mice were quantified by RT-qPCR with normalization against 18S rRNA. Data were expressed as means ± s.d. (*n* = 4) and analyzed by two-tailed unpaired *t*-test. *P < 0.05, **P < 0.01. **j** Fortilin blocks apoptosis and IRE1α pathway activation in EGF-SubA-challenged liver. Paraffin sections from the livers of EGF-SubA-treated fortilin^WT-liver^ and fortilin^KO-liver^ mice were subjected to TUNEL, P-IRE1α, and P-JNK staining using 3,3’-diaminobenzidine (DAB) as a chromogen. Data were expressed as means ± s.d. (*n* = 6) and analyzed by two-tailed unpaired *t*-test. ***P < 0.005. Scale bar = 50 µm

To study how protection by fortilin against the activation of the IRE1α pathway manifests itself in the whole animal under ER stress, we generated a mouse strain that lacks fortilin specifically in the liver—*alb*-Cre^+/−^fortilin^flox/flox^(fortilin^KO-liver^)—by crossing transgenic mice overexpressing the albumin-promoter-driven Cre recombinase (*alb*-Cre^+/−^) with fortilin^flox/flox^ mice (Supplementary Fig. 4A, B, C). These mice appeared normal and were fertile. The liver of the fortilin^KO-liver^ mice expressed very little fortilin (Fig. 4a, top panel; Supplementary Fig. 8), whereas fortilin expression was similar in other organs between fortilin^KO-liver^ and fortilin^WT-liver^ mice (Fig. 4a, bottom panel). We then intraperitoneally administered a one-time dose of EGF-SubA (250 µg kg^−1^ body weight) to fortilin^KO-liver^ and fortilin^WT-liver^ mice. The liver expresses the EGF receptor and thus responds to EGF-SubA^43^.

The first group of animals (*n* = 10 per group) were observed for 48 days to assess survival (Fig. 4b, the left arm, “Survival”), whereas the animals in the second group were sacrificed on day 7, and the blood and organs were harvested for further analyses (Fig. 4b, the right arm, the mechanism of action or “MOA”). In the survival experiment, 80% of fortilin^WT-liver^ mice were still alive on day 48, whereas none of the fortilin^KO-liver^ mice survived (all died between days 9 and 12) (*P* < 0.001 by the Kaplan–Meier survival curve and log-rank test, Fig. 4c). In the MOA group (*n* = 10 each), the complete metabolic panel (CMP) showed a drastic elevation of liver enzymes—both alanine aminotransferase (ALT) and alkaline phosphatase (ALP)—in fortilin^KO-liver^ mice compared with fortilin^WT-liver^ mice, suggesting that fortilin^KO-liver^ mice sustained far worse liver injury than did fortilin^WT-liver^ mice (Fig. 4d, ALT and ALP). Consistently, the liver function of fortilin^KO-liver^ mice was more seriously compromised than that of fortilin^WT-liver^ mice upon EGF-SubA challenge, as evidenced by higher total bilirubin levels (Fig. 4d, TBIL). There was no significant difference between EGF-SubA-challenged fortilin^WT-liver^ and fortilin^KO-liver^ mice in hemoglobin/hematocrit (H/H) or white blood cell (WBC) counts (Supplementary Table 2). Upon gross inspection, the livers of fortilin^KO-liver^ mice were uniformly pale, indicating severe functional impairment of the organ (Fig. 4e, EGF-SubA of fortilin^KO-liver^). The kidney and pancreas were not affected by EGF-SubA challenge (Supplementary Fig. 4D, blood urea nitrogen [BUN] and creatinine [CRE] for renal function and amylase [AMY] for pancreas). These data suggest that fortilin^KO-liver^ mice died of acute liver failure in response to EGF-SubA challenge.

### Fortilin blocks IRE1α from signaling apoptosis in the liver

We then tested whether fortilin negatively regulated IRE1α in vivo, as we saw in the cellular and in vitro systems described above (Figs. 1, 2, and 3). Immunoblot analyses showed that fortilin expression was severely decreased in the livers of fortilin^KO-liver^ mice regardless of treatment—PBS or EGF-SubA (Fig. 4f, g, row a, lanes 1–4 vs. lanes 5–8; Supplementary Fig. 8). There was no difference between the livers of EGF-SubA-challenged fortilin^KO-liver^ and fortilin^WT-liver^ mice in the levels of P-PERK, PERK, P-eIF2α, eIF2α, and ATF6f (Fig. 4f, IB and the bottom graph). In contrast, the phosphorylation of IRE1α (Fig. 4g, bands b2, b3, and b4 vs. bands b6, b7, and b8, and also the bottom graph) and JNK (Fig. 4g, bands d2, d3, and d4 vs. bands d6, d7, and d8, and also the bottom graph; and Supplementary Fig. 4E for ELISA of JNK and P-JNK) in the liver was more extensive in EGF-SubA-treated fortilin^KO-liver^ mice than in EGF-SubA-treated fortilin^WT-liver^ mice. The processing of *XBP1* into the active form *XBP1s* by P-IRE1α was also greater in the livers of fortilin^KO-liver^ mice than in those of fortilin^WT-liver^ mice (Fig. 4h, lanes 2–6 vs. lanes 8–12 for EGF-SubA-treated fortilin^WT-liver^ and fortilin^KO-liver^ mice, respectively, also the bottom graph).

Because XBP1s, a transcriptional factor, induces genes involved in ER-associated degradation (ERAD)^9^, we tested whether *Edem1* and *Herp1*—ERAD genes—are induced more robustly in the livers of fortilin^KO-liver^mice than in fortilin^WT-liver^ mice in response to EGF-SubA challenge. We performed quantitative reverse transcription polymerase chain reaction (RT-qPCR) on the total liver RNA and found that both *Edem1* and *Herp1* were induced more in the livers of EGF-SubA-treated fortilin^KO-liver^ mice than in those of EGF-SubA-treated fortilin^WT-liver^ mice (Fig. 4i).

Hematoxylin and eosin (H&E) staining of SubA-treated liver sections also showed more drastic histopathological changes in fortilin^KO-liver^ mice than in fortilin^WT-liver^ mice (Supplementary Fig. 4F, total injury score = 17 vs. 14, respectively). Terminal deoxynucleotidyl transferase dUTP nick end labeling (TUNEL) staining of the liver sections showed that the livers of fortilin^KO-liver^ mice challenged by EGF-SubA exhibited a higher degree of apoptosis than those of fortilin^WT-liver^ mice (Fig. 4j, TUNEL; ***P < 0.005 by two-tailed unpaired *t*-test). Consistent with the immunoblot analysis results (Fig. 4g, rows b and d), immunostaining showed higher levels of P-IRE1α and P-JNK in the livers of fortilin^KO-liver^ mice than in those of fortilin^WT-liver^ mice upon EGF-SubA challenge (Fig. 4j, P-IRE1α, P-JNK; ***P < 0.005 by two-tailed unpaired *t*-test). TUNEL positivity and the immunoreactivity of P-IRE1α and P-JNK in the PBS-treated livers of fortilin^KO-liver^ and fortilin^WT-liver^ mice were equally low (Supplementary Fig. 4G). These data suggest that fortilin in the liver protects whole animals against ER stress-induced liver damage and death by mitigating the activation of the IRE1α stress sensing pathway and hepatocyte apoptosis.

### Fortilin blocks apoptosis in the liver by inhibiting IRE1α

**Fig. 5:**
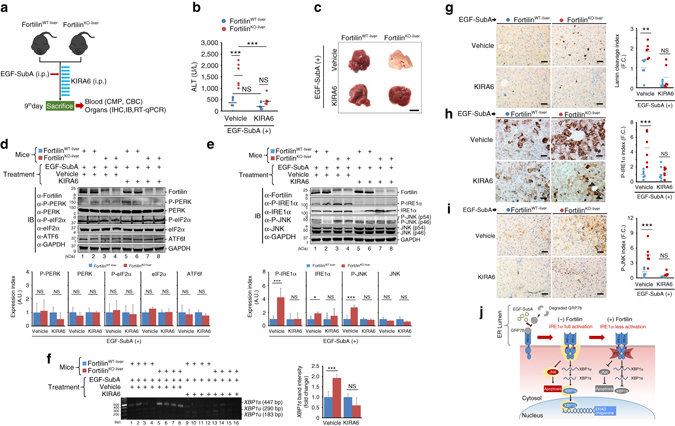
Protection by fortilin against EGF-SubA-induced liver damage is mediated by its ability to block the activation of the IRE1α-JNK apoptosis pathway. **a** Experimental protocol. Five-week-old male fortilin^WT-liver^ or fortilin^KO-liver^ mice (*n* = 6) were pretreated by vehicle or the IRE1α kinase inhibiting RNAse attenuator-6 (KIRA6) for 3 days, challenged by EGF-SubA once on the third day, and treated by vehicle or KIRA6 for an additional 6 days. **b** KIRA6 protects both fortilin^WT-liver^ and fortilin^KO-liver^ mice against EGF-SubA-induced liver damage. The sera from EGF-SubA-challenged fortilin^WT-liver^ and fortilin^KO-liver^ mice, treated with either vehicle or KIRA6, were assayed for ALT. Data were expressed as means ± s.d. (*n* = 6) and analyzed by two-tailed unpaired *t*-test. *NS* not statistically significant; ***P < 0.005. **c** The livers of EGF-SubA-challenged fortilin^WT-liver^ and fortilin^KO-liver^ mice exhibit similar gross appearance when treated with KIRA6. Scale bar = 10 mm. **d** Lack of change in expression and phosphorylation patterns of the PERK and ATF6 pathway proteins in the EGF-SubA-challenged livers, regardless of the status of fortilin or of KIRA6 treatment. The total lysates from the livers of EGF-SubA-challenged fortilin^WT-liver^ and fortilin^KO-liver^ mice, treated with either KIRA6 or vehicle, were subjected to quantitative IB using the indicated antibodies. Data were expressed as means ± s.d. (*n* = 4) and analyzed by two-tailed unpaired *t*-test. **e** The livers of EGF-SubA-challenged fortilin^WT-liver^ and fortilin^KO-liver^ mice exhibit a similar degree of IRE1α and JNK phosphorylation when treated with KIRA6. Data were expressed as means ± s.d. (*n* = 4) and analyzed by two-tailed unpaired *t*-test. *P < 0.05; ***P < 0.005. **f** The livers of EGF-SubA-challenged, KIRA6-treated, fortilin^WT-liver^ and fortilin^KO-liver^ mice exhibit a similar degree of *XBP1* splicing. The total RNA from the livers of EGF-SubA-challenged fortilin^WT-liver^ and fortilin^KO-liver^ mice were assayed for the amounts of *XBP1s* and *XBP1u*. Data were expressed as means ± s.d. (*n* = 4) and analyzed by two-tailed unpaired *t*-test. ***P < 0.005. **g** The livers of EGF-SubA-challenged fortilin^WT-liver^ and fortilin^KO-liver^ mice exhibit equal amounts of apoptosis when treated with KIRA6. Paraffin sections from the livers of EGF-SubA-treated fortilin^WT-liver^ and fortilin^KO-liver^ mice (*n* = 6 each) for cleaved lamin. Data were expressed as means ± s.d. (*n* = 6) and analyzed by two-tailed unpaired *t*-test. **P < 0.01; *F.C*. fold change. Scale bar = 50 µm. **h**, **i** The immunostaining of the livers of EGF-SubA-challenged fortilin^WT-liver^ and fortilin^KO-liver^ mice exhibit the same degree of IRE1α pathway activation when treated with KIRA6. Data were expressed as means ± s.d. (*n* = 6) and analyzed by two-tailed unpaired *t*-test. ***P < 0.005. Scale bar = 50 µm. **j** Proposed model of the role of fortilin in ER stress-induced apoptosis

We demonstrated that the lack of fortilin in the liver sensitizes hepatocytes to EGF-SubA-induced, ER stress-mediated activation of IRE1α pathway (as evidenced by the phosphorylation of both IRE1α and JNK, Fig. 4g, rows b and d) and apoptosis (as evidenced by increased TUNEL positive hepatocytes, Fig. 4j). Because fortilin is an anti-apoptotic protein^16, 20–22^, however, we still do not know whether the apoptosis of fortilin-deficient hepatocytes is the result of (i) EGF-SubA-induced hyperactivation of the IRE1α pathway in the absence of fortilin or (ii) activation of other apoptotic pathways that are normally and negatively regulated by fortilin. To evaluate the exact role of fortilin in EGF-SubA-induced ER stress-mediated liver damage, we repeated the same experiment in the presence of the specific IRE1α inhibitor KIRA6^44^ (Fig. 5a). KIRA6 (CAS Registry Number = 1589527-65-0, MW = 518.5) is an imidazopyrazine-based small-molecule IRE1α inhibitor that competitively binds the ATP binding site of the kinase domain (Fig. 3c) and blocks both the kinase and RNase activities of the molecule^45^. We pretreated fortilin^WT-liver^ and fortilin^KO-liver^ mice with KIRA6 for 3 days, injected EGF-SubA (250 µg kg^−1^ body weight) once intraperitoneally on the third day, continued KIRA6 treatment for another 6 days, sacrificed the animals on the ninth day, and subjected the blood to CMP and complete blood count (CBC) and the livers to histological and molecular analyses. All animals survived for 9 days. Consistent with data shown in Fig. 4d, when treated with the vehicle (not KIRA6), fortilin^KO-liver^ mice showed a drastically higher elevation of ALT than did fortilin^WT-liver^ mice (Fig. 5b, Vehicle). When treated with KIRA6, however, neither the ALT (Fig. 5b, KIRA6) nor the ALP/TBIL (Supplementary Fig. 5) levels of EGF-SubA-challenged fortilin^KO-liver^ mice were statistically significantly different from those of fortilin^WT-liver^mice. There was no significant difference in H/H or WBC counts between EGF-SubA-challenged fortilin^WT-liver^ and fortilin^KO-liver^ mice, regardless of KIRA6 treatment (Supplementary Table 3). Upon gross inspection, the livers of fortilin^KO-liver^ mice appeared similar to those of fortilin^WT-liver^ mice when treated with KIRA6 (Fig. 5c, KIRA6). Mechanistically, KIRA6 treatment did not change the way the PERK and ATF6 pathways responded to EGF-SubA in the presence and absence of fortilin in the liver (Fig. 5d; Supplementary Fig. 8). Although IRE1α was more phosphorylated in the livers of vehicle-treated fortilin^KO-liver^ mice than in those of vehicle-treated fortilin^WT-liver^ mice (Fig. 5e, bands b3 and b4 vs. bands b1 and b2, respectively; Supplementary Fig. 8), the degree of the phosphorylation of IRE1α in the livers of KIRA6-treated fortilin^KO-liver^ mice was similar to and as low as that in the livers of KIRA6-treated fortilin^WT-liver^ mice (Fig. 5e, bands b7 and b8 vs. b5 and b6, respectively). In addition, although JNK was more phosphorylated in the livers of vehicle-treated fortilin^KO-liver^ mice than in those of vehicle-treated fortilin^WT-liver^ mice (Fig. 5e, bands d3 and d4 vs. bands d1 and d2, respectively), the degree of the phosphorylation of JNK in the livers of KIRA6-treated fortilin^KO-liver^ mice and was similar to that in the livers of KIRA6-treated fortilin^WT-liver^ mice (Fig. 5e, bands d7 and d8 vs. d5 and d6, respectively). Further, when treated with KIRA6, the processing of *XBP1* into the active form *XBP1s* was no longer significantly different between fortilin^WT-liver^ and fortilin^KO-liver^ mice (Fig. 5f, lanes 9–12 vs. lanes 13–16; also the right panel). Moreover, when treated with KIRA6, the livers of fortilin^KO-liver^ mice challenged by EGF-SubA exhibited the same degree of lamin cleavage (i.e., apoptosis) as those of fortilin^WT-liver^ mice (Fig. 5g). Finally, consistent with these immunoblot analysis results (Fig. 5e, rows b and d), immunostaining showed the same levels of P-IRE1α and P-JNK in the livers of fortilin^KO-liver^ mice and fortilin^WT-liver^ mice when the EGF-SubA-challenged mice were treated with KIRA6 (Fig. 5h, i). Taken together, these data suggest that fortilin blocked the majority of EGF-SubA-mediated liver damage—as evidenced by elevated ALT (Fig. 5b), ALP (Supplementary Fig. 5), and TBIL (Supplementary Fig. 5) levels—and apoptosis (Fig. 5g) through its specific inhibition of the IRE1α pathway.

## Discussion

The current work reveals yet another facet of the anti-apoptotic activity of fortilin. Previous studies have shown that fortilin exerts its anti-apoptotic activity through (a) its binding to and stabilization of MCL1^21^, a BCL-2 family member and macrophage survival factor^46^, (b) binding to and de-stabilization of transforming growth factor-β-stimulated clone-22 (TSC-22), a pro-apoptotic protein^47^, (c) binding to Ca^2+^ and blockade of Ca^2+^-dependent apoptosis^48^, and (d) binding to and inhibition of the tumor suppressor protein p53^22^. Based on the current work, we now know that fortilin also inhibits apoptosis by directly interacting with IRE1α in its cytosolic domain and blocking the activation of its downstream pathways—XBP1 and JNK (the latter leading to apoptosis). Importantly, fortilin selectively blocks the IRE1α pathway without affecting the PERK and ATF6 pathways (Figs. 2d–f, 4f–h, and 4j). Our current working model is as follows (Fig. 5j): Upon ER stress, IRE1α molecules, unphosphorylated and monomeric at the baseline^2^, form clusters with each other and start to trans-autophosphorylate each other^2^. As soon as IRE1α becomes phosphorylated (P-IRE1α), fortilin strongly binds to it (Fig. 3a, b, d, e, and Supplementary Fig. 3A) and prevents it from phosphorylating other IRE1α molecules (Fig. 2e, row b; Fig. 3l; Supplementary Fig. 3F; Fig. 4g, row b; Fig. 4j, P-IRE1α; Fig. 5e, row b). The fact that fortilin binds P-IRE1α (Fig. 3a, the right panel, lanes 3 and 4; Fig. 3e) and not IRE1α (Fig. 3a, the right panel, lanes1 and 2; Fig. 3d) does not necessarily contradict the fact that fortilin decreases IRE1α phosphorylation, because P-IRE1α is the very kinase that phosphorylates IRE1α (trans-autophosphorylation). P-IRE1α, which is the activated form of IRE1α, activates both the JNK apoptosis pathway (Fig. 2e, row d) and the XBP1 pathway (Fig. 2e, row f; Fig. 2f) in a dose-dependent fashion. Importantly, our molecular docking data (Fig. 3i, j) suggest that the binding of fortilin to P-IRE1α prevents the molecule from gaining access to and phosphorylating the other IRE1α, as fortilin obliterates the kinase core of the activated P-IRE1α (Fig. 3i). The MD simulation data (Fig. 3j) support the notion that fortilin preferentially binds to P-IRE1α because the fortilin–P-IRE1α complex is more thermodynamically stable than the fortilin–IRE1α complex.

The UPR is designed to achieve two distinct biological goals: (i) the preservation of stressed cells through restoration of ER homeostasis and (ii) the elimination of afflicted cells through apoptosis when they are damaged beyond repair. No ER stress trigger is known to exert either homeostatic or apoptotic UPR alone^49^. Rather, ER stress activates all UPR signaling pathways and simultaneously produces both homeostatic and apoptotic outputs^49^. How does fortilin participate in a life-or-death decision of ER-stressed cells whose IRE1α pathway is activated? Because fortilin negatively regulates both JNK (pro-apoptotic) and XBP1 (ER homeostasis, pro-survival) pathways through its binding to and inhibition of P-IRE1α (Fig. 5j), the anti-apoptotic activity of fortilin could be overcome by its inability to mount sufficient pro-survival signals, leading to apoptotic death of the cells. Experimentally, however, fortilin consistently protected the cells (Fig. 1c–h, Supplementary Fig. 1D, E, Fig. 4j [TUNEL], and 5g) against apoptosis. A hint may lie in the fact that *XBP1* mRNA splicing activity is only transiently activated in the early phase of the UPR^49, 50^ and that fortilin is gradually induced by various stresses—such as high concentration glucose^51^, heat^52^, and ROS^53^—and remains upregulated for a longer duration than does *XBP1* mRNA splicing activity. In the early phase of the UPR when fortilin exists at a lower concentration in the cell, the *XBP1* pathway might promote cell survival by restoring its homeostasis. In the late phase of the UPR when the *XBP1* mRNA splicing activity subsides^49, 50^, fortilin might sustain cell survival by blocking the JNK pro-apoptotic pathway. Further investigation is needed to understand how fortilin navigates the dichotomy between JNK-induced apoptosis and XBP1-mediated homeostasis and survival.

Although many proteins interact with IRE1α^42^ (Supplementary Fig. 3G), only three are known to bind IRE1α and negatively regulate the IRE1α pathway aside from fortilin. They are BAX inhibitor 1 (BI-1, also known as transmembrane Bax inhibitor motif containing 6 [TMBIM6])^54^, Jun activation domain-binding protein-1 (JAB1, also known as CSN5 and COPS5)^55^, and receptor for activated C-kinase 1 (RACK1, also known as guanine nucleotide binding protein, beta polypeptide 2-like 1 [GNB2L1])^56^ (Supplementary Fig. 3G,﻿*). BI-1 is a 295-aa transmembrane protein that binds to the cytosolic portion of IRE1α and inhibits the processing of *XBP1* mRNA. JAB1, a 334-aa protein with a nuclear localization signal, binds to the linker region of the cytosolic portion of IRE1α in the absence of stress. Upon ER stress, JAB1 dissociates from IRE1α, allowing IRE1α to splice *XBP1* mRNA into *XBP1s*, suggesting that JAB1 inhibits IRE1α through its binding^55^. It is unknown how the association between JAB1 and IRE1α is regulated or whether JAB1 prevents the kinase activity of IRE1α. RACK1, a 317-aa scaffold protein containing seven Trp-Asp 40 repeats, binds to the linker region of IRE1α^56^. RACK1 also binds to protein phosphatase 2A (PP2A), positions IRE1α and PP2A close to each other, and keeps IRE1α dephosphorylated and inactivated^56^. ER stress increases the association between RACK1 and IRE1α, and PP2A dissociates from RACK1 under ER stress, facilitating autophosphorylation and activation of IRE1α^56^. In the current work, we found that fortilin, a 172-aa protein with no homology to BI-1, JAB1, or RACK1, also binds to the cytosolic portion of IRE1α. Unlike JAB1, which dissociates from IRE1α in response to ER stress, fortilin translocates from the nucleus to the cytosol upon ER stress, gains access to the cytosolic portion of IRE1α (Fig. 1a and Supplementary Fig. 1A), and preferentially binds to P-IRE1α (Fig. 3a, e, j). There, unlike BI-1, JAB1, or RACK1, fortilin uniquely inhibits both the kinase (Fig. 2e, row d; Figs. 3l and 4g, row d; Fig. 4j, P-JNK; Supplementary Figs. 2 and 4E) and RNase (Fig. 2e, row f; Figs. 2f, 3k, 4h, i) activities of IRE1α.

Rapidly growing tumors may outgrow their vascular supply and experience hypoxia and hypoglycemia^2^. For tumor cells to adapt to and survive in this adverse microenvironment, activation of the UPR is crucial^57^. In fact, the activation of IRE1α has been shown to facilitate angiogenesis and invasion of cancerous cells^58^. Fortilin, an inhibitor of activated IRE1α (Fig. 5j) may inhibit IRE1α-mediated tumor growth and invasion in certain tumors. There has been no effort made to test whether fortilin is mutated and made dysfunctional in certain tumors. It is possible that certain fortilin mutations could make fortilin unable to bind IRE1α, thus activating the XBP1 homeostasis branch of the IRE1α pathway and facilitating tumor growth and propagation. In contrast, other tumors could take advantage of wild-type fortilin to block the activation of the IRE1α-JNK apoptosis pathway to survive and propagate.

Hepatocytes, which produce and secrete a large amount of proteins, are naturally susceptible to ER stress. ER stress has been shown to contribute to various liver diseases^59^. Results of the current study show that the lack of fortilin made the liver unusually susceptible to ER stress-induced dysfunction and injury (Figs. 4 and 5 and Supplementary Figs. 4 and 5). It is likely that fortilin would fortify the liver against ER stress-induced and IRE1α-mediated liver injury, which is known to occur in alcoholic liver disease^60^ and viral hepatitis^61^.

Further studies are needed to therapeutically exploit the newly unraveled physical and functional interaction between fortilin and IRE1α in human diseases. The fortilin^flox/flox^ mice described herein (Supplementary Fig. 4A, B) should be a viable translational tool to evaluate the role of fortilin and the fortilin-IRE1α interaction in handling stress in a tissue-specific fashion.

## Methods

### Molecular cloning

For cloning of fortilin complementary DNA (cDNA) into the pESG-IBA5-vector, the cDNA encoding human fortilin obtained previously in our laboratory^16^ was cloned into the multiple cloning site of the pESG-IBA5 mammalian expression vector (IBA Life Sciences, Göttingen, Germany) using a PCR-based strategy.

### Cell culture and cell lines

Both U2OS (Catalog #: HTB-96) and PC3 (Catalog #: CRL-1435) cell lines were obtained from American Type Culture Collection (ATCC, Manassas, VA, USA). These cell lines were maintained in high-glucose Dulbecco’s modified Eagle’s medium and Roswell Park Memorial Institute (RPMI) 1640 medium, respectively, and supplemented with 10% fetal bovine serum (FBS) (HyClone, Logan, UT, USA) at 37 °C in an atmosphere containing 5% CO_2_.MycoFluor™ (ThermoFisher Scientific-Molecular Probe, Eugene, OR, USA) was used to detect mycoplasma contamination when appropriate.

U2OS cells stably overexpressing fortilin (U2OS_Fortilin-HA_) or influenza hemagglutinin (HA) epitope-tag sequence YPYDVPDYA only (U2OS_Empty-HA_) were generated as described previously^62^. To generate the fortilin-deficient and control PC3 cell lines (PC3_sh-Fortilin_ and PC3_sh-Empty_), PC3 cells were transduced with the lentiviral particles containing the short hairpin RNA (shRNA) against human fortilin (Lentivirus^sh-Fortilin^) and Lentivirus^sh-Empty^, which were generated, according to the manufacturer’s instructions, from the pLKO.1-puro vector containing the shRNA against human fortilin (pLKO.1-puro-sh-Fortilin) and the empty shRNA sequence (pLKO.1-puro-sh-Empty) (Sigma-Aldrich, St Louis, MO, USA), respectively, selected under puromycin (2.5 μg mL^−1^), and characterized by immunoblot analysis. The cell lines were maintained in RPMI supplemented with 10% FBS and puromycin (2.5 µg mL^−1^, Mediatech, Inc., Manassas, VA, USA).

### Immunoblot analyses

SDS-PAGE and immunoblot analyses were performed as described previously^34^. The following primary antibodies were used at the indicated dilutions/concentrations: Mouse anti-ATF6 (dilution used = 1:250, Clone 70B1413.1, Imgenex-Novus Biologicals, Littleton, CO, USA); Mouse anti-α-tubulin (dilution used = 1:250, Clone B-7, Santa Cruz Biotechnology, Dallas, TX, USA); Mouse anti-β-actin (dilution used = 1:250, Clone C4, Santa Cruz Biotechnology); Goat anti-calnexin (dilution used = 1:250, ﻿sc-6465, Santa Cruz Biotechnology); Rabbit anti-calreticulin (dilution used = 1:1000, Clone D3E6, #12238, Cell Signaling Technologies, Danvers, MA, USA); Rabbit anti-caspase-3 (dilution used = 1:1000, Clone 8G10, Cell Signaling Technologies), which was used to detect full-length, p17, and p19 caspase-3; Rabbit anti-caspase-9 (dilution used = 1:1000, #9502, Cell Signaling Technologies), which was used to detect full-length and cleaved caspase-9; Rabbit anti-cytochrome c (dilution used = 1:1000, Clone 136F3, Cell Signaling Technologies); Rabbit anti-eIF2α (dilution used = 1:500, #9722, Cell Signaling Technologies); Rabbit anti-phospho-human-eIF2α (dilution used = 1:500, Ser^51^, #9721, Cell Signaling Technologies); Rabbit anti-phospho-mouse-eIF2α (dilution used = 1:1000, Ser^51^, Clone D9G8, Cell Signaling Technologies); Mouse anti-fortilin (dilution used = 1:1000, Clone M03, Abnova, Taipei City, Taiwan), which was used for all experiments other than those shown in Supplementary Fig. 1A and Supplementary Fig. 1B; Rabbit anti-fortilin (dilution used = 1:250, PM017, MBL International, Woburn, MA, USA), which was used for the experiments depicted in Supplementary Fig. 1A and B; Rabbit anti-fortilin monoclonal antibody (concentration used = 14 µg mL^−1^, Clone EPR5540, ab133568, Abcam), which was used to immunoprecipitate fortilin, as shown in Fig. 3b and Supplementary Fig. 3A; Mouse anti-GAPDH (dilution used = 1:10,000, Clone 10R-G109a, Fitzgerald, Acton, MA, USA); Rabbit anti-GRP78 (dilution used = 1:500, sc-13968, Santa Cruz Biotechnology); Mouse anti-HDAC2 (dilution used = 1:1000, Clone Y461, Abcam); Rabbit anti-HSP60 (dilution used = 1:1000, Clone D6F1, Cell Signaling Technologies); Rabbit anti-HSP90 (dilution used = 1:1000, #4874, Cell Signaling Technologies); Rabbit anti-IRE1α (dilution used = 1:500, sc-20790, Santa Cruz Biotechnology); Rabbit anti-phospho-IRE1α (dilution used = 1:1000, ab48187, Abcam); Rabbit anti-JNK (dilution used = 1:500, sc-571, Santa Cruz Biotechnology); Rabbit anti-phospho-human-JNK (dilution used = 1:500, #9251, Cell Signaling Technologies); Rabbit anti-phospho-mouse-JNK (dilution used = 1:250, 07-175, EMD Millipore, Billerica, MA, USA); Rabbit anti-cleaved Lamin A (dilution used = 1:250, #2035, Cell Signaling Technologies); Rabbit anti-MBP (dilution used = 1:500, sc-13914, Santa Cruz Biotechnology); Mouse anti-NQO2 (dilution used = 1:250, Clone A-5, Santa Cruz Biotechnology); Rabbit anti-PARP (dilution used = 1:1000, #9542, Cell Signaling Technologies), which was used to detect full-length PARP; Rabbit anti-cleaved PARP (Asp^214^) (dilution used = 1:1000, Clone D64E10, #5625, Cell Signaling Technologies); Rabbit anti-human-PERK (dilution used = 1:500, #5683, Cell Signaling Technologies); Rabbit anti-mouse-PERK (dilution used = 1:500, sc-13073, Santa Cruz Biotechnology); Mouse anti-phosphoserine (dilution used = 1:1000, Clone 4A4, EMD Millipore); Rabbit anti-XBP1 (dilution used = 1:500, sc-7160, Santa Cruz Biotechnology). All antibodies were used with appropriate IRDye680LT- or IRDye800CW-conjugated secondary antibodies (LI-COR, Lincoln, NE, USA). The signal intensities of protein bands were quantified using the Odyssey Infrared Imaging System (LI-COR).

### Subcellular fractionation

ER subcellular fractions were obtained from 1.5 × 10^6^ PC3 cells treated with either 0.5 µM TG, 2 nM EGF-SubA, or dimethyl sulfoxide (DMSO) using the Subcellular Protein Fractionation kit (Pierce, Rockford, IL, USA) according to the manufacturer’s instructions. Protein concentrations of each fraction were determined using BCA methods (Bio-Rad, Hercules, CA, USA). Exactly 10 μg of total proteins were resolved by 12% SDS-PAGE and subjected to immunoblot analysis using anti-calreticulin (an ER marker^63^), anti-HSP90 (cytosol marker^64^), HDAC2 (a nuclear marker^65^), and anti-fortilin antibodies.

### DNA fragmentation assay

The Cell Death Detection ELISA PLUS kit (Roche, Indianapolis, IN, USA) was used following the manufacturer’s instructions, with modifications described previously^22^. For Fig. 1c, d, PC3_sh-Fortilin_ or PC3_sh-Empty_ cells (0.3 × 10^6^ per well) were seeded into six-well plates. The next morning, cells were treated with various concentrations of TG (0, 0.5, and 1.0 µM) or EGF-SubA (0.2, 2, and 20 nM, SibTech, Brookfield, CT, USA) for 24 h before they were harvested (both adherent and floating) and subjected to the same assay^22^. For Supplementary Fig. 1D, U2OS_Empty-HA_ or U2OS_Fortilin-HA_ cells (0.3 × 10^6^ per well) were seeded into six-well plates. The next morning, cells were treated with DMSO or 0.5 µM TG for 24 h before they were harvested (both adherent and floating) and subjected to the assay^22^.

### Flow cytometric analysis of apoptotic and necrotic cells

To assess the relative contribution of apoptosis and necrosis to total cell death induced by TG and EGF-SubA, PC3 cells were stained with FITC-conjugated Annexin V and PI (1 µg mL^−1^) and subjected to flow cytometry analysis per the manufacturer’s instructions (ThermoFisher-Invitrogen-Molecular Probes). The population separated into four groups: (i) live cells without apoptosis or necrosis (no red or green fluorescence), (ii) apoptotic cells without necrosis (positive green fluorescence and negative red fluorescence), (iii) necrotic cells without apoptosis (negative green fluorescence and positive red fluorescence), and (iv) cells exhibiting both necrosis and apoptosis (positive green and red fluorescence). Flow cytometry data are represented as mean ± s.d. of four independent experiments.

### Cytochrome c release assay

The cytosolic and membrane fractions from PC3_sh-Fortilin_ and PC3_sh-Empty_ cells stimulated by 1.0 µM and 20 nM of TG and EGF-SubA, respectively, for 24 h were obtained using the Subcellular Protein Fractionation Kit for cultured cells (Catalog #: 78840, ThermoFisher Scientific) according to the manufacturer’s instructions. Fifty microgram of cytosolic and membrane (mitochondrial) fraction protein extracts were loaded onto each lane of a 12% SDS-polyacrylamide gel, separated, and blotted to nitrocellulose membranes, which were probed by anti-cytochrome c antibody, anti-HSP90 (cytosolic marker protein^64^), and anti-HSP60 (mitochondrial marker protein^64^).

### Immunocytochemistry of fortilin and the ER marker protein disulfide isomerase (PDI)

Immunocytochemical analyses to determine subcellular localization of fortilin under ER stress were performed using the SelectFX Alexa Fluor 488 Endoplasmic Reticulum Labeling kit (Molecular Probes) as previously described^16^. U2OS cells were seeded on a cover glass, fixed in 4% buffered formalin solution for 5 min, permeabilized in 0.2% Triton X, and incubated with rabbit anti-fortilin (MBL International) and mouse anti-PDI (Molecular Probes) antibodies. After washes, bound antibodies were detected with goat anti-rabbit AlexaFluor® 568-conjugated and goat anti-mouse AlexaFluor® 488-conjugated (Invitrogen, Grand Island, NY, USA) secondary antibodies, respectively. 4′,6-diamidino-2-phenylindole (DAPI, Sigma-Aldrich) was used to counterstain the nuclear DNA. The stained slides were examined under a fluorescence microscope (Eclipse Ti, Nikon Inc., Melville, NY, USA) with appropriate filter sets. The extent of fortilin recruitment to the ER was quantified by counting cells (at least 50) and determining the percentage of cells that exhibit co-localization of AlexaFluor® 568 and AlexaFluor® 488 signals; results are expressed as % co-localization (Supplementary Fig. 1A).

### MTT cell survival assay

The assay was performed as previously described^66^. U2OS_Empty-HA_ or U2OS_Fortilin-HA_ cells were plated in 96-well plates (1 × 10^4^ cells per well). The next day, cells were challenged with either 0.5 µM TG or DMSO, treated with 10 µg mL^−1^ of 3-(4,5-dimethylthiazol-2-yl)-2,5-diphenyltetrazolium bromide (MTT, Sigma-Aldrich) for 4 h, and solubilized by solubilization buffer (10% SDS in 0.01 N HCl) overnight. Formed MTT formazan was quantified by measuring the absorbance of each well at 600 nm (A_600_) using a SpectraMax spectrophotometer (Molecular Devices, Sunnyvale, CA, USA). The cell survival rate was quantified as follows: (A_600_ of treated cells−A_600_ of background)/(A_600_ of untreated cells–A_600_ of background) × 100; results are expressed as arbitrary units (A.U.) (Supplementary Fig. 1C).

### Caspase activity assays

Assays to measure caspase-3, -8, and -9 activities of U2OS_Empy-HA_ and U2OS_Fortilin-HA_ cells treated for 6 h with either DMSO for TG were performed as described previously^16^ and using fluorometric assay kits according to the manufacturer’s instructions (Biovision, Mountain View, CA, USA).

### XBP splicing assay

Analysis of *XBP1* splicing as a surrogate marker of IRE1α activation was performed as previously described^67^. Briefly, first-strand cDNA was synthesized from the total RNA isolated from PC3 cells (Fig. 2f) or mouse livers (Figs. 4h and 5f) using SuperScript III reverse transcriptase (Invitrogen). Because activated IRE1α processes the *XBP1* mRNA (*XBP1u*, 473 base pairs (bp)) to a shorter *XBP1* mRNA that lacks a 26 bp fragment including the *PstI* restriction site (*XBP1s*, 447 bp), cDNA from the spliced form *XBP1s* is resistant to *PstI*, whereas that from the unprocessed form *XBP1u* is digested by *PstI* into 290 and 183 bp fragments. The cDNAs of *XBP1u* and *XBP1s* were amplified by PCR using the same primer set: 5′-AAACAGAGTAGCAGCGCAGACTGC-3′ and 5′- TCCTTCTGGGTAGACCTCTGGGAG -3′. The amplicons were digested by *PstI*, resolved on 2% agarose gels containing ethidium bromide, and then visualized using the Gel Doc XR + system (Bio-Rad). The 447 bp band represented the cDNA amplicon from the processed *XBP1s*, whereas both the 290 and 183 bp bands originated from the cDNA amplicon from the unprocessed *XBP1u*. The band density was quantified using the Gel Doc™ XR + System (Bio-Rad), and results are expressed as A.U.

### Enzyme-linked immunosorbent assay

Quantifications of total JNK and P-JNK in EGF-SubA-challenged PC3_sh-Fortilin_ and PC3_sh-Empty_ cells as well as EGF-SubA-treated livers of fortilin^WT-liver^ and fortilin^KO-liver^ mice were achieved using the PathScan® Total SAPK/JNK Sandwich ELISA Kit and PathScan® Phospho-SAPK/JNK (Thr183/Tyr185) Sandwich ELISA Kit, respectively (Cell Signaling Technology). For the cell-based assay, PC3_sh-Fortilin_ and PC3_sh-Empty_ cells were plated into six-well plates (0.3 × 10^6^ cells per well), treated with EGF-SubA (0–20 nM, SibTech) for 24 h, and subjected to ELISA. For the whole animals, fortilin^WT-liver^, and fortilin^KO-liver^ mice were intraperitoneally administered 250 µg kg^−1^ body weight of EGF-SubA and sacrificed on day 7. The total lysates from the livers were then subjected to ELISA according to the manufacturer’s instructions.

### Proximity ligation assay

PLA was originally described by Soderberg et al.^31^. Wild-type PC3 cells were seeded on a chamber slide, treated with either PBS or 2 µM EGF-SubA (SibTech), fixed in 10% buffered formalin solution, permeabilized in 0.1% Triton X, and incubated with (a) mouse anti-fortilin (2C4, Abnova) and (b) either rabbit anti-IRE1α (Santa Cruz Biotechnology) or rabbit anti-phospho-IRE1α (Abcam) antibodies. The chamber slide was then incubated for 1 h with secondary anti-mouse and anti-rabbit antibodies conjugated to oligonucleotides (PLA probes MINUS and PLUS, Duolink In Situ Proximity Ligation Assay, Sigma-Aldrich) before ligase and two connector oligonucleotides were added to the solution. These oligonucleotides hybridize to the two PLA probes and join them into a closed circle if they are in close proximity (about 30 nm). Subsequently, fluorescently labeled oligonucleotides that hybridized to the rolling circle amplification product were added. The nuclei were stained with DAPI. A Zeiss LSM 510 Meta confocal microscope system (Oberkochen, Germany) was used to visualize the signals. The PLA Interaction Index was calculated by dividing the number of red dots by the number of nuclei as stained by DAPI; results are expressed as A.U.

### Immunoprecipitation and co-immunoprecipitation

Cleared total cell lysates from PC3 cells treated with 2 nM EGF-SubA for 24 h were incubated with either rabbit monoclonal anti-fortilin antibody (Clone EPR5540, Abcam) or rabbit IgG. Formed protein–antibody complexes were precipitated by Dynabeads™ Protein G (Dynal-ThermoFisher Scientific), washed four times, eluted into SDS gel loading buffer; and subjected to SDS-PAGE, immunoblot transfer, and immunodetection. Successful immunoprecipitation of fortilin was confirmed using anti-fortilin (M03, Abnova) antibody, whereas anti-IRE1α, anti-P-IRE1α, anti-β-actin, and anti-α-tubulin antibodies were used to detect co-immunoprecipitated IRE1α, P-IRE1α, β-actin, and α-tubulin, respectively. Both β-actin and α-tubulin have been shown to interact with fortilin^32, 33^. NQO2, a protein that is known not to interact with fortilin^34^, was used as negative control to ensure that sufficient washing to eliminate non-specific bindings occurred. Rabbit IgG was used to rule out the non-specific interaction that withstood the wash conditions used in the protocol.

### Generation of recombinant human fortilin

Affinity purification of human recombinant fortilin was performed using the Strep-tag purification system (IBA Life Sciences) as described previously^68^. 293T cells stably expressing human fortilin tagged with the Strep-tag II (WSHPQFEK) at its N-terminal end were collected by trypsinization, washed in PBS, resuspended in buffer W (100 mM Tris HCl [pH = 8], 150 mM NaCl, 1 mM EDTA), lysed by repeated freeze-thaw cycles, and sonicated to shear the genomic DNA. Cleared total cell lysate was then passed through a column packed with Strep-Tactin-Superflow resin (IBA Life Sciences). The column was washed five times with Buffer W before the Strep-tagged fortilin was eluted with Buffer E (Buffer W plus 2.5 mM desthiobiotin). The fractions were pooled and concentrated using centrifugal filters (Amicon® EMD Millipore). The concentrated protein samples were buffer-exchanged into PBS using Zeba™ Spin Desalting Columns (ThermoFisher Scientific). The final purification product was characterized by Coomassie and immunoblot analyses (Supplementary Fig. 3B, lane 9; Supplementary Fig. 3F, fortilin).

### Dephosphorylation of P-IRE1α

Dephosphorylation of recombinant P-IRE1α was performed using calf intestinal alkaline phosphatase (CIP)-agarose beads according to the manufacturer’s instructions (Sigma-Aldrich). Recombinant phospho-IRE1α (0.4 μg protein per sample, Origene, Rockville, MD, USA) was resuspended in reaction buffer (50 mM Tris HCl, 10 mM NaCl, 1 mM MgCl_2_, 0.1 mM dithiothreitol (DTT), pH 7.9) and treated with CIP-agarose (1 unit of CIP per 0.4 μg of protein) at 37 °C for 2 h. The reaction was stopped by separating the bead-bound enzyme from the reaction mixture by centrifugation at 1902 × *g*. The CIP-treated sample was subjected to immunoblotting using rabbit anti-phospho IRE1α antibody (Abcam) (Supplementary Fig. 3E).

### Bio-layer interferometry

Recombinant human fortilin protein produced as described above was biotinylated and immobilized on streptavidin-coated biosensors (ForteBio, Menlo Park, CA, USA) at a concentration of 25 μg mL^−1^ for 600 s, followed by buffer exchange in PBS. The system was tested using anti-fortilin antibody (positive control, Abnova) and bovine serum albumin (negative control, Sigma-Aldrich) as depicted in Supplementary Fig. 3C, D. Next, various concentrations of recombinant human phopho-IRE1α-Myc-DDK (aa 1–977, Origene), de-phosphorylated IRE1α-Myc-DDK (aa 1–977, Origene, see above for the dephosphorylation procedure), GST-IRE1α-L (aa 1–70, Abnova), GST-IRE1α-TM (aa 401–500, Abnova), and GST-IRE1α-C (aa 468–977, SignalChem, Richmond, British Columbia, Canada) were added for 180 s to evaluate the association between the two molecules. The integrity of these recombinant proteins was evaluated by SDS-PAGE and Coomassie staining (Supplementary Fig. 3B, lanes 2, 5, 6, 7, and 9). Finally, the reaction mixture was replaced with PBS for 300 s to evaluate the dissociation. The binding data were processed and the dissociation constant (Kd) was calculated using BLItz analysis software (ForteBio). The data depicted in Fig. 3d–h and Supplementary Fig. 3C, D represent the results of three independent binding experiments.

### Molecular docking assay of fortilin and IRE1α

To study how fortilin and IRE1α interact with each other, we performed a molecular docking experiment using Piper software on the ClusPro Server 2.0^69^ and the crystal structures of fortilin (2HR9) and IRE1α (4U6R) available from the Protein Data Bank^70^. We evaluated docking results using clustering analysis and weighted model scores as described by Kozakov et al.^71^ From the top 10 cluster populations, we selected the binding pose that yielded the best average cluster weighed score and used it for the determination of binding free-energy by MD. For MD simulations, we used the Gromacs v5.0.4 software package utilizing the GROMOS 54A7 force-field parameter set^72^. We adopted force-field parameters for post-translational modification of phosphorylation from Hansson et al.^73^ and applied them to the GROMOS 54A7 force field as previously described^72^. An initial 100 ns MD simulation of fortilin bound with IRE1α was performed using the binding configuration obtained from the protein-protein docking studies. Simulations were performed using periodic boundary conditions with explicit solvation using a simple point charge model potential. To prepare the system for simulation, a conjugate gradient energy minimization followed by NVT (number of particle, volume, and temperature) and NPT (number of particle, pressure, and temperature) equilibrations were performed. A 100 ps equilibration was conducted under a NVT ensemble using a v-rescale thermostat at 300 K with a coupling time constant of 0.1 ps. A subsequent 200 ps NPT equilibration was performed using the isotropic Parrinello-Rahman barostat with a time constant of 1.0 ps. The integration time steps for both equilibration steps were 1 fs. The 100 ns MD simulation was executed using 2 fs time steps. All bonds were constrained to equilibrium values using the LINCS algorithm^74^. The Verlet cutoff scheme was used in calculation of short-range Coulomb and van der Waals forces, and particle-mesh Ewald was used for long-range electrostatics. The averaged structure over the final 10 ns was used in the subsequent free-energy simulations. Phosphoserine groups were added to IRE1α at S^724^ and S^726^, followed by energy minimization and equilibration of the system as described above. To calculate the relative binding free energy of fortilin between phosphorylated (P-IRE1α) and unphosphorylated IRE1α (IRE1α), the following classic thermodynamic cycle was used: where Δ*G*_1_ is the free energy change resulting from phosphorylation of IRE1α at S^724^ and S^726^ (P-IRE1α) in the unbound state and Δ*G*_2_ is the free energy change resulting from phosphorylation of IRE1α in a bound state with fortilin. Δ*G*_3_ and Δ*G*_4_ are the free energies of binding of fortilin with IRE1α and P-IRE1α, respectively. To determine the relative binding free energy of fortilin between phosphorylated and unphosphorylated IRE1α (Δ*G*_3_−Δ*G*_4_), the following relationship can be used: ΔΔ*G* = Δ*G*_3_−Δ*G*_4_ = Δ*G*_1_−Δ*G*_2_. Free energy calculations were performed to compute the free energy change due to phosphorylation of IRE1α in the unbound and bound states, representing Δ*G*_1_ and Δ*G*_2_, respectively. Simulation of IRE1α phosphorylation (and reverse simulation of dephosphorylation) was implemented in two steps constituting three states: A, B, and C. State A represents phosphorylated IRE1α, state B corresponds to an intermediate conformation for which the partial charges on the phosphate moiety are set to zero, and state C represents unphosphorylated IRE1α, where the partial charges and van der Waals radii of the atoms of the phosphate moiety are set to zero. Calculations were performed for λ values at non-equidistant nodes provided in Supplementary Table 4. The partial charges of modified atoms were decreased from *λ* = 0 to *λ* = 1 through seven intermediate *λ* values. The van der Waals parameters were decreased from *λ* = 0 to *λ* = 1 through seven intermediate steps. During decoupling of van der Waals interactions, soft-core potential functions were applied with *α*_LJ_ = 0.5 and *λ* power dependency set to 1. At each *λ* value, simulations were performed for 500 ps at 0.5 fs time steps. ∂H/∂λ was saved every 10 fs for post-processing and free-energy calculations using Bennett’s acceptance ratio (BAR) perturbation method^75^. Only the last 250 ps of each simulation were used for BAR calculations, the first 250 ps being considered as additional system equilibration.

### In vitro IRE1α RNase activity assay

The endoribonuclease (RNase) activity of IRE1α in the presence of fortilin was assayed in vitro using fluorescence resonance energy transfer (FRET)-based methods described previously^39^. Briefly, recombinant human IRE1α (10 µg, SignalChem) was pre-incubated with various concentrations of recombinant human fortilin (0–4 µM) in RNase Assay Buffer (50 mM HEPES, pH 7.2, 100 mM KOAc, 0.005% TritonX-100, 5 mM DTT, 5% glycerol) for 30 min at room temperature. Next, 25 µl of 2 µM fluorescently tagged *XBP1* RNA stem loop (5′-FAM-CAGUCCGCAGCACUG-Iowa-Black-FQ-3′, Integrated DNA Technologies, Coralville, IA, USA) were added to the reaction mixture, which was then incubated for 90 min at room temperature. Fluorescence was read using a SpectraMax M2e plate reader (Molecular Devices) with excitation and emission at 495 and 520 nm, respectively.

### In vitro IRE1α kinase activity assay

The kinase activity of IRE1α in the presence of fortilin was assayed in vitro as previously described^41^ using (a) [γ-^33^P]ATP or (b) anti-phosphoserine antibody as follows. First recombinant human IRE1α (15 nM, SignalChem), MBP (20 µM, SignalChem), and various concentrations of fortilin were prepared in kinase reaction buffer (20 mM HEPES, pH 7.5, 10 mM MgCl_2_, 1 mM EGTA, 0.02% Brij35, 0.02 mg mL^−1^ BSA, 0.1 mM Na_3_VO_4_, 2 mM DTT, and 1% DMSO) and incubated for 20 min at room temperature. The reaction was initiated by addition of [γ-^33^P]ATP (10 µM), carried out for 2 h at room temperature, and terminated by spotting the reaction mixture onto phospho-cellulose P81 ion exchange filter paper (GE Whatman, Pittsburgh, PA, USA). The filter paper was washed three times with 1% phosphoric acid to remove unbound phosphate, placed in a scintillation tube containing scintillation cocktail, and subjected to scintillation counting. After subtracting background derived from control reactions lacking the kinase, kinase activity data were expressed as the percent kinase activity in test samples compared to reaction mixture with no fortilin. The data from two independent experiments were interpolated to a sigmoidal four parameter logistic curve, from which the half maximal inhibitory concentration (IC_50_) of fortilin was derived (GraphPad Prism, La Jolla, CA, USA) (Fig. 3l). For the further assessment of in vitro IRE1α kinase activity, recombinant human IRE1α (0.5 µg, SignalChem) was pre-incubated with various concentrations of recombinant human fortilin (0–4 µg) in Kinase Assay Buffer (25 mM MOPS pH 7.2, 12.5 mM β-glycerol-phosphate, 25 mM MgCl_2_, 5 mM EGTA, 2 mM EDTA, 0.25 mM DTT, 20 µM ATP) for 15 min at room temperature, followed by the addition of 2 µg of MBP (SignalChem) as a universal kinase substrate^76^ to the reaction mixture. The reaction mixture was incubated at 30 °C for 45 min and subjected to SDS-PAGE and immunoblot analysis using anti-phosphoserine (EMD Millipore), anti-MBP (Santa Cruz), anti-fortilin (Abnova), and anti-phospho-IRE1α (Abcam) antibodies. The phosphorylation index was calculated by dividing the signal intensity of phosphorylated MBP by that of total MBP; results are expressed as A.U. Two independent experiments were performed with consistent findings (Supplementary Fig. 3F).

### Generation of liver-specific fortilin knockout mice

Fortilin^flox/flox^ mice—in which the fortilin gene was flanked by the LoxP sequence to allow tissue-specific deletion—were generated using the standard homologous recombination technique as previously described^77^. First, a mouse BAC clone containing the full fortilin gene was isolated from the C57BL/6J ES BAC clone library. A fortilin targeting vector was constructed by subcloning (a) the 4.8 kilobase (kb) upstream genomic sequence, (b) the 3.6 kb genomic DNA sequence containing all six fortilin exons (exon 1–exon 6), and (c) the 3.2 kb downstream genomic DNA sequence into the pVBFRTCKR targeting vector (Supplementary Fig. 4A, #2) that already contained (a) two LoxP sequences, (b) a phosphoglycerate kinase promoter-driven neomycin resistance gene flanked by two flippase recognition target sequences, and (c) the thymidine kinase cassette. After extensive sequencing to verify the integrity of the construct, the targeting vector was linealized by I-CeuI (R0699S, New England BioLabs, Ipswich, MA, USA) and electroporated into C57BL6 embryonic stem cells (ESCs). ESC clones that survived in neomycin-containing media were analyzed by PCR and Southern blotting (using radioactive DNA probes 1 and 2 after NdeI [R0111S, New England BioLabs] and NsiI [R0127S] digestion, respectively) for successful homologous recombination (Supplementary Fig. 4B). Two mutated ESC lines (B43 and B61) were microinjected into C57BL6 blastocysts, which were then implanted in pseudopregnant host mice to obtain chimeric mice. Chimeric mice were then crossed with C57BL6 mates, and their offspring were tested using PCR-based methods for germline transmission. The resultants fortilin^flox/flox^ mice were fully in the C57BL/6J genetic background from the beginning. Fortilin^flox/flox^ mice were then crossed with a transgenic flipase strain to remove the neomycin resistance gene cassette. For the generation of liver-specific fortilin KO mice, fortilin^floxflox^ mice were crossed with C57BL/6J mice overexpressing the Cre recombinase transgene under the control of albumin enhancer/promoter (Stock #: 0003574, Jackson Laboratory, Bar Harbor, ME, USA) to generate liver-specific deletion of fortilin. Because the expression levels of fortilin in the livers of the Alb-Cre^+/−^fortilin^flox/flox^ mice were found substantively lower than their control Alb-Cre^−/−^fortilin^flox/flox^ mice (Fig. 4a), we performed all subsequent experiments using Alb-Cre^+/−^fortilin^flox/flox^ (denoted fortilin^KO-liver^ hereafter) mice, without having to generate Alb-Cre^+/+^fortilin^flox/flox^ mice. The actual production of fortilin^KO-liver^ and fortilin^WT-liver^ (Alb-Cre^−/−^fortilin^flox/flox^) mice for the experiments was achieved by mating Alb-Cre^+/−^fortilin^flox/flox^ and Alb-Cre^−/−^fortilin^flox/flox^ mice, yielding fortilin^KO-liver^ and fortilin^WT-liver^ mice in a 1:1 ratio. Genotyping of fortilin^KO-liver^ and fortilin^WT-liver^ mice was performed on tail-derived genomic DNA using standard PCR-based methods. The presence of the Cre recombinase transgene was detected using the following primer sets: 5′-GCGGTC TGGCAGTAAAAACTATC-3′ (Supplementary Fig. 4A, M1) and 5′-GTGAAACAGCATTGCTGTCACTT-3′ (Supplementary Fig. 4A, M2), where Alb-Cre^+/−^ mice yielded a 100 bp amplified fragment and Alb-Cre^−/−^ mice yielded no amplicons. The presence of the LoxP-fortilin-LoxP knock-in construct was verified using the following primer sets: 5′-TGGACC CTGACTTTCATCACCTC-3′ (Supplementary Fig. 4A, F1) and 5′-GTCATCTAACCTTACCCCAGTAAGC-3′ (Supplementary Fig. 4A, F2), where fortilin^flox/flox^ mice yielded a 405 bp fragment and wild-type fortilin mice (fortilin^WT/WT^ mice) yielded a 280 bp fragment.

### Mouse model of ER stress-induced liver damage

For EGF-SubA treatment, 5-week-old male fortilin^KO-liver^ and fortilin^WT-liver^ mice were intraperitoneally administered either PBS or EGF-SubA (Sibtech). For survival analyses, mice were kept on a 12 h dark–light cycle and had access to food and water ad libitum. They were examined daily for their behavior and health. For the mechanism of action (MOA) analyses, mice were sacrificed by CO_2_ inhalation and cervical dislocation 7 days after injection, and whole blood and organs were collected for further analyses. There was no blinding performed. For KIRA6 treatment, 5-week-old male fortilin^KO-liver^ and fortilin^WT-liver^ mice were intraperitoneally administered either 5 mg kg^−1^ KIRA6 (Catalog #: 532281, EMB Millipore) in solution made of 3% ethanol, 7% Tween-80, 90% saline or the same solution without KIRA6 as vehicle daily for 9 days. On day 3 of the experiment, mice were intraperitoneally injected with 250 μg kg^−1^ of EGF-SubA (SibTech) once. Mice were examined daily for their behavior and health for a total of 9 days before they were sacrificed. The whole blood and organs were collected for further analyses (*n* = 4 each for immunoblot and XBP splicing assays, *n* = 6 for CBC, CMP, and immunohistochemistry) as described below. There was no blinding performed.

### Whole-blood analyses

For the CMP and CBC, whole blood was sampled by cardiocentesis using a 25-gauge needle immediately after euthanasia by CO_2_ and cervical dislocation. The sample was transferred into Microtainer® tubes with lithium heparin (BD Biosciences, Franklin Lakes, NJ, USA) by removing the needle from the syringe, pouring the blood into the tube, and mixing it thoroughly with the lithium heparin. The blood chemistry profile was obtained using the Comprehensive Diagnostic Panel rotors and the Vetscan VS2 (Abaxis, Union City, CA, USA). The following blood chemistry parameters were assayed for each sample: albumin (ALB), ALP, ALT, AMY, TBIL, BUN, total calcium (Ca^2+^), phosphorus (PHOS), CRE, glucose (GLU), sodium (Na^+^), potassium (K^+^), total protein (TP), and globulin (GLOB). The results are shown in Figs. 4d and 5b and Supplementary Figs. 4D and 5. CBC was obtained using the HEMAVET 950FS Hematology System (Drew Scientific, Dallas, TX, USA). The CBC results are shown in Supplementary Tables 2 and 3.

### Quantitative reverse transcription polymerase chain reaction

RT-qPCR was performed as described previously^22^. Briefly, the livers of fortilin^WT-liver^ and fortilin^KO-liver^ mice were harvested into Tri-Reagent (Molecular Research Center, Cincinnati, OH, USA). RNA was isolated in accordance with the manufacturer’s instructions and treated with DNAse (ABI, Foster City, CA, USA). RT-qPCR was performed in quadruplicate with exactly 50 ng of total RNA using the TaqMan® RT-PCR kit (Applied Biosystems [ABI] at Life Technologies, Grant Island, NY, USA) in the ABI Step One Plus Real-Time PCR system and the following primer and probe sets (Integrated DNA Technologies): Mouse *Edem1*—forward: 5′-TCTGGTTGATGCCTTGGATAC-3′, reverse: 5′-GACCTGGACTGTGGAATCTTT-3′, probe 5′-FAM-CCGAGTTCC/ZEN/AGAAGGCAGTCAAGTT-IAbkFQ-3′ where FAM = carboxyfluorescein, IAbkFQ = Iowa Black FQ, and ZEN™ = an internal quencher to enhance the quenching activity of the 3′ quencher Iowa Black FQ; Mouse *Herp1*—forward: 5′-CACCTTGTGTGCAATGTGAAG-3′, reverse: 5′-CCAGGATGCTGTGTCTGATT-3′, probe: 5′-FAM-AATGCCAGA/ZEN/AACCAGCACAAAGGG-IAbkFQ-3′; and Mouse 18S ribosomal RNA (rRNA)—forward: 5′-GCCGCTAGAGGTGAAATTCT-3′, reverse: 5′-TCGGAACTACGACGGTATCT-3′, probe: 5′-JOEN-ACCAGAGCG/ZEN/AAA GCATTTGCCAAG-IAbkFQ-3′ where JOEN = 6-carboxy-4′,5′-dichloro-2′,7′-dimethoxyfluorescein.

### Hematoxylin and eosin staining

The formalin-fixed H&E sections of liver tissue were evaluated on day 7 post-SubA challenge for histopathologic changes. The various parameters assessed included steatosis (macro- and micro-vesicular), apoptosis, mitosis, inflammation, pattern of injury (for example, zones 1, 2, or 3), and markers of injury such as the presence of giant mitochondria or Mallory’s hyaline. The sections (*n* = 6 each for SubA-treated fortilin^WT-liver^ and fortilin^KO-liver^ mice; *n* = 3 for PBS-treated fortilin^KO-liver^ mice; *n* = 2 for PBS-treated fortilin^WT-liver^ mice) were blindly evaluated by an experienced hepatopathologist, who graded the above features on a scale of 0 to 4. Apoptotic and mitotic counts were assessed semi-quantitatively by counting the number per a high-power field.

### Immunohistochemistry

All immunostained sections were digitally imaged using a Nikon CoolScope (Nikon). Immunohistochemistry of mouse liver was performed as described previously^78^ using (a) anti-phospho-IRE1α (Abcam), (b) anti-phospho-JNK (ThermoFisher Scientific), and (c) anti-cleaved lamin (small subunit, Cell Signaling Technology) antibodies and 3,3′-diaminobenzidine (DAB) as the chromogen. Phospho-IRE1α, phospho-JNK, and cleaved lamin A indices were calculated by dividing the DAB-positive area by the region of interest (ROI), and results are expressed as A.U. The cleavage of lamin is a well-characterized event in apoptosis^79^. TUNEL staining was performed as previously described^17, 22^ using the FragEL^TM^ DNA Fragmentation Detection Kit (EMD Millipore-Calbiochem) in accordance with the manufacturer’s instructions. TUNEL-positive cells within 0.145 mm^2^ of the ROI were counted, and TUNEL indices were calculated as the number of TUNEL-positive cells per unit area (in mm^2^); results are expressed as A.U.

### Ethics statement

This study was carried out in accordance with the recommendations in the Guide for the Care and Use of Laboratory Animals of the National Institutes of Health. All experiments involving animals were approved by the Institutional Animal Care and Use Committees.

### Statistical analysis

The degree of the spread of data was expressed by the standard deviation (± s.d.). Two-tailed unpaired *t*-test was used to compare the means of two groups. *P* < 0.05 was considered to be statistically significant. The numbers of mice used in in vivo experiments were determined by (i) power analysis, assuming *α* error rate of 0.05, *β* error rate of 0.20, and expected difference of 25% and using Minitab 17 (State College, PA, USA) or (ii) our previous data set and experience from the similar experiments performed in the past.

### Data availability

The authors declare that the data supporting the findings of this study are available within the paper and its Supplementary Information files. All relevant data are available from the authors upon request.

## Electronic supplementary material

Supplementary Information Supplementary figures and supplementary tables
