# Supplementary material for: Fortilin binds IRE1α and prevents ER stress from signaling apoptotic cell death
Source: Nat Commun. 2017 May 26;8:18. doi: 10.1038/s41467-017-00029-1 (PMC5446404; doi:10.1038/s41467-017-00029-1)
Supplement: Supplementary file 1 — Supplementary figures and supplementary tables [file 41467_2017_29_MOESM1_ESM.pdf]

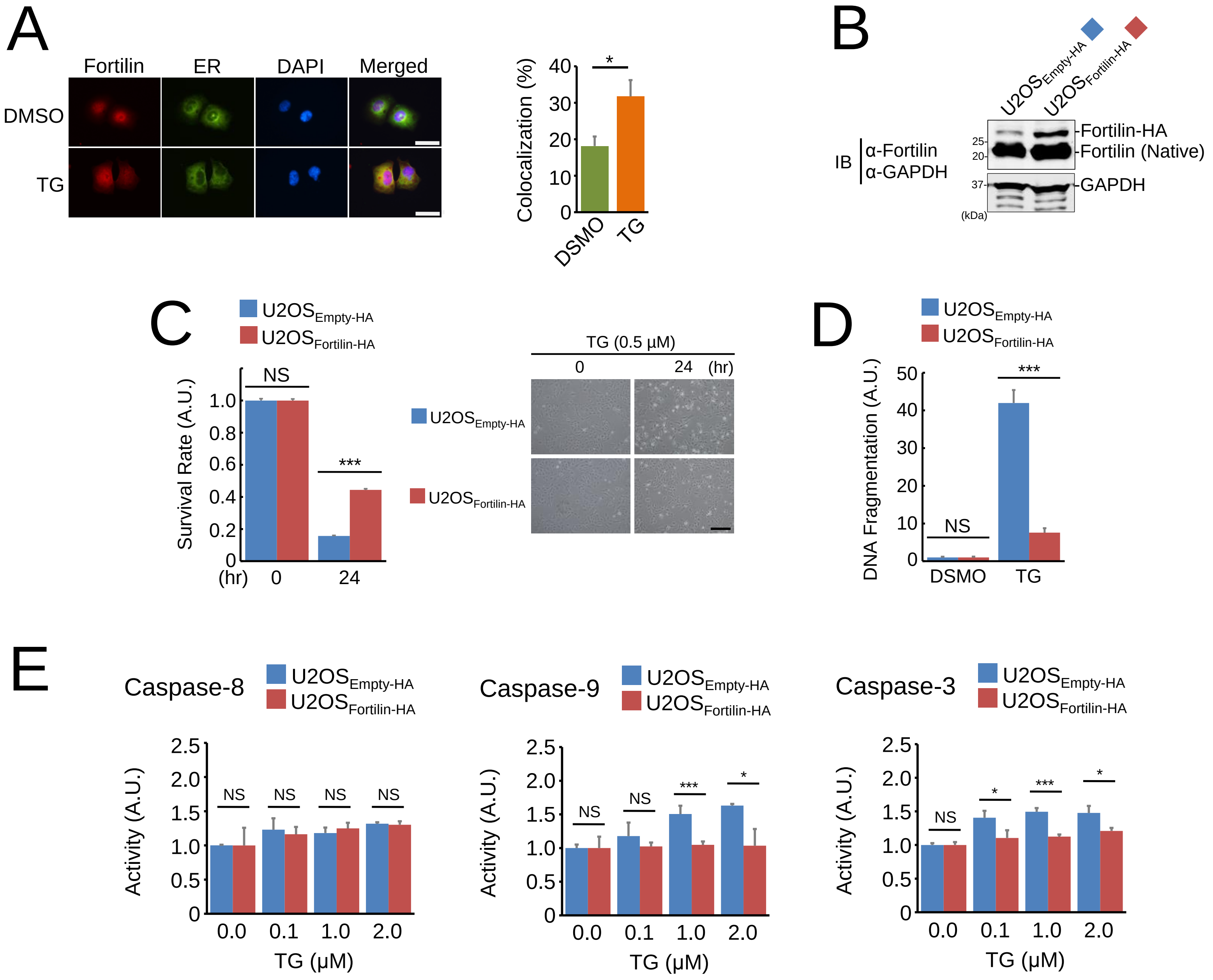

Supplementary Fig. 1. **Fortilin protects cells against ER stress-induced apoptosis.** Abbreviations: DMSO, dimethyl sulfoxide; TG, thapsigargin; ER, endoplasmic reticulum; DAPI, 4',6-diamidino-2-phenylindole; IB, immunoblot; A.U., arbitrary units; α-GAPDH, anti-glyceraldehyde 3-phosphate dehydrogenase antibody; $U2OS_{Empty\text{-}HA}$, U2OS cells overexpressing influenza hemagglutinin (HA) epitope-tag sequence YPYDVPDYA; $U2OS_{Fortiln\text{-}HA}$, U2OS cells overexpressing HA-tagged fortilin; Data were expressed as means ± s.d. The number of biological replicates was denoted as n. Statistical analyses were performed using two-tailed unpaired t-test. *NS*, not statistically significant; *, $P < 0.05$; ***, $P < 0.005$ (See also Fig. 1.) (**A**) Fortilin recruitment to the ER region upon ER stress as assessed by immunocytochemistry. U2OS cells, stimulated by DMSO or TG (n =3), were stained for fortilin, the ER marker protein disulfide isomerase, and the nuclear DNA. Bar graphs show the percentages of cells showing fortilin co-localized with the ER from three independent experiments. Scale bar = 50 µm. Taken together with the data in Fig. 1A, results show that fortilin is translocated from the nucleus to the cytosol and recruited to the cytosolic surface of the ER. (**B**) Generation and characterization of $U2OS_{Empty\text{-}HA}$ and $U2OS_{Fortilin\text{-}HA}$ cells. U2OS cells stably overexpressing fortilin-HA ($U2OS_{Fortilin\text{-}HA}$) and HA-tag ($U2OS_{Empty\text{-}HA}$) were generated and characterized by immunoblot analysis. (**C**) Protection by fortilin against ER stress-induced cell death. $U2OS_{Empty\text{-}HA}$ and $U2OS_{Fortilin\text{-}HA}$ cells were challenged by TG and their survival rates were assessed by MTT assays (n = 4) and phase-contrast microscopy. Scale bar = 250 µm. (**D**) Anti-apoptotic activity of fortilin in cells under ER stress. The degree of DNA fragmentation was assayed in $U2OS_{Empty\text{-}HA}$ and $U2OS_{Fortilin\text{-}HA}$ cells treated with either DMSO or TG. Bar graphs show the DNA fragmentation levels of $U2OS_{Empty\text{-}HA}$ and $U2OS_{Fortilin\text{-}HA}$ cells (n = 3) (**E**) Inhibition of activation of caspases -9 and -3 by fortilin in cells under ER stress. $U2OS_{Empty\text{-}HA}$ and $U2OS_{Fortilin\text{-}HA}$ cells were treated with the indicated concentrations of TG for 6 h before being subjected to caspase-8, -9, and -3 activity assays. Bar graphs show the respective caspase activities (n = 3).

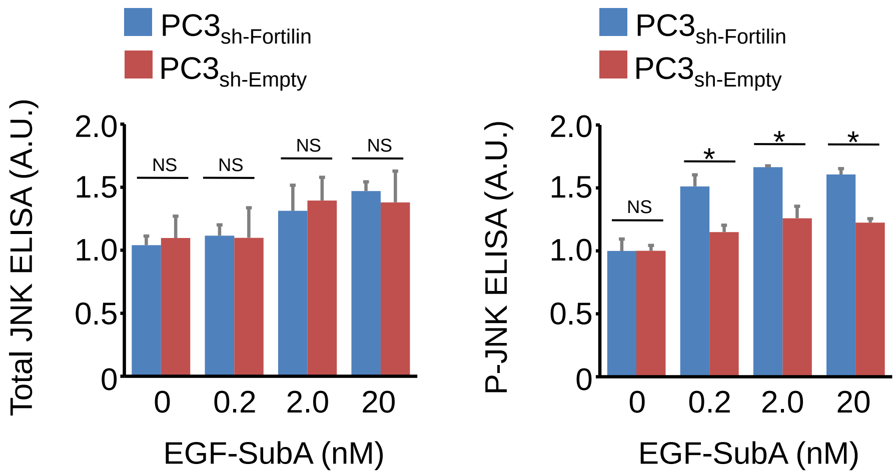

Supplementary Figure 2. **Fortilin inhibits ER stress-induced phosphorylation of JNK.** Abbreviations: EGF-SubA, the epidermal growth factor (EGF) fused to the proteolytic A subunit of the bacterial AB_5 toxin (SubA); ELISA, enzyme-linked immunosorbent assay; $PC3_{sh\text{-}Fortilin}$, PC3 cells with fortilin knockdown; $PC3_{sh\text{-}Empty}$, PC3 cells harboring empty shRNA silencing lentiviral vector; A.U., arbitrary units; P-JNK, phosphorylated JNK. Data were expressed as means ± s.d. and analyzed by two-tailed unpaired t-test. NS, not statistically significant; *, $P < 0.05$. (See also Fig. 2.) $PC3_{sh\text{-}Empty}$ and $PC3_{sh\text{-}Fortilin}$ cells were treated with the indicated concentrations of EGF-SubA before their lysates were subjected to ELISA to determine the concentrations of total JNK and P-JNK. Biological replicates = 3.

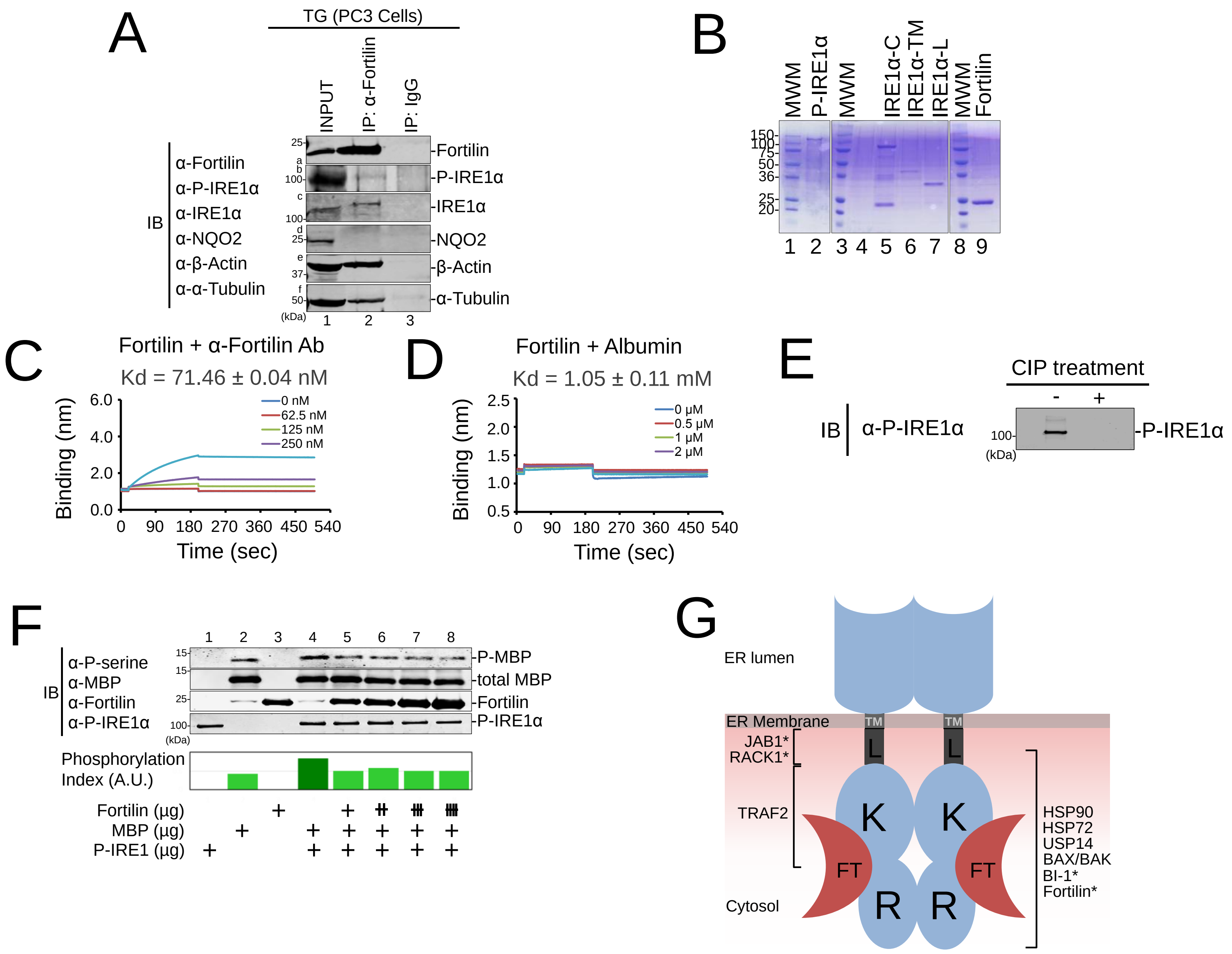

Supplementary Figure 3. **Fortilin interacts with IRE1α in TG-stimulated PC3 cells and inhibits its kinase activities.** Abbreviations: MWM, molecular weight marker; IRE1α-L, GST-IRE1α (aa 1–70); IRE1α-TM, GST-IRE1α (aa 401–500); IRE1α-C, GST-IRE1α (aa 468–977); Kd, dissociation constant; CIP, calf intestinal phosphatase; ER, endoplasmic reticulum; K, the kinase domain of IRE1α, R, the RNase domain of IRE1α; FT, fortilin; JAB1, Jun activation domain-binding protein-1; RACK1, receptor for activated C-kinase 1; TRAF2, TNF receptor associated factor 2; HSP90, heat shock protein 90; HSP72, heat shock protein 72; USP14, ubiquitin specific peptidase 14; BAX, BCL2 associated X; BAK, BCL2 antagonist killer 1; BI-1, BAX inhibitor 1. (See also Fig. 3.) (**A**) Immunoprecipitation-Western blot analysis of fortilin and its protein partners. The cleared total cell lysates from TG-challenged human prostate cancer PC3 cells were subjected to immunoprecipitation by rabbit anti-fortilin monoclonal antibody or rabbit IgG. Formed protein-antibody complexes were precipitated by Dynabeads™ Protein G before they were resolved by SDS-PAGE, transferred to the nitrocellulose membranes, and probed by the indicated antibodies. Fortilin co-immunoprecipitated P-IRE1α, IRE1α, β-actin, and α-tubulin but not NQO2. (**B**) Coomassie staining of recombinant IRE1αs and fortilin. Recombinant IRE1α, including IRE1α-L, GST-IRE1α (aa 1–70), IRE1α-TM, GST-IRE1α (aa 401–500); IRE1α-C, GST-IRE1α (aa 468–977), and fortilin were resolved in SDS-PAGE and visualized by Coomassie staining to verify their structural integrity. Lane 4 is a blank lane. (**C, D**) Specific binding of fortilin to α-fortilin antibody and the lack of fortilin binding to albumin in bio-layer interferometry. Biotinylated fortilin was immobilized to the streptavidin biosensor. To confirm the proper functioning of the system, α-fortilin antibody (positive control) and albumin (negative control) were applied to the biosensor at various concentrations and Kds were calculated. Experiments were repeated three times, and the depicted sensograms are representative of them. Kds were expressed as mean ± s.d. and calculated from the

data from three independent experiments. Fortilin binds α-fortilin antibody at the Kd of 71.46 ± 0.04 nM but does not bind albumin (Kd = 1.05 ± 0.11 mM). (**E**) Successful dephosphorylation of P-IRE1α by CIP. Recombinant phosphorylated IRE1α was incubated with CIP-agarose beads. CIP-agarose was removed from the reaction mixture by centrifugation. The reaction mixture was subjected to Western blotting and immunodetection using rabbit anti-phospho IRE1α antibody. (**F**) Inhibition by fortilin of the kinase activity of IRE1α. An in vitro IRE1α kinase activity assay was performed by incubating IRE1α with recombinant fortilin and MBP as a substrate of the kinase. Anti-phosphoserine antibody was used to quantify the amount of phosphorylated MBP in the presence of fortilin. The phosphorylation index was calculated by dividing the signal intensity of phosphorylated MBP (P-MBP) by that of total MBP and is expressed as arbitrary units (A.U.). Three independent experiments were performed with consistent findings. (**G**) IRE1α-binding proteins. Proteins that have been shown to bind the cytosolic portion of the IRE1α are shown. The asterisk (*) denotes the molecules that bind and negatively regulate IRE1α (JAB1, RACK1, BI-1, and fortilin). JAB1 and RACK1 bind to the linker portion of the IRE1α; TRAF2 binds to the kinase domain; HSP90, HSP72, USP14, BAX/BAK, BI-1, and fortilin bind to somewhere in the cytosolic domain (consisting of the linker portion, kinase domain, and RNase domain) of the IRE1α. See text for details.

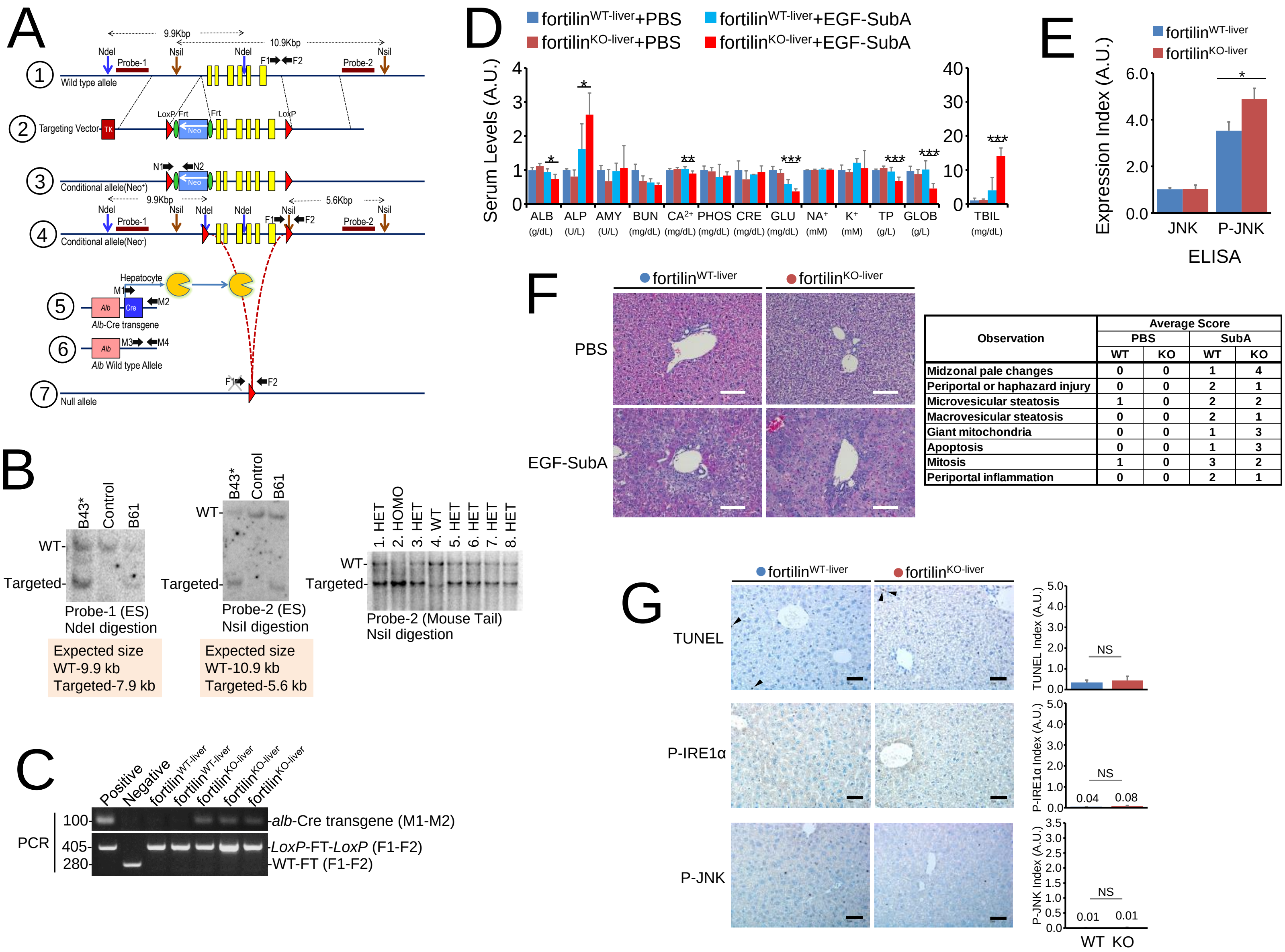

Observation	Average Score			
	PBS		SubA	
	WT	KO	WT	KO
Midzonal pale changes	0	0	1	4
Periportal or haphazard injury	0	0	2	1
Microvesicular steatosis	1	0	2	2
Macrovesicular steatosis	0	0	2	1
Giant mitochondria	0	0	1	3
Apoptosis	0	0	1	3
Mitosis	1	0	3	2
Periportal inflammation	0	0	2	1

Supplementary Figure 4. **Mice lacking fortilin in the liver were generated, characterized, and found more susceptible to SubA-induced ER stress-mediated liver injury than did wild-type control mice.** Abbreviations: TK, thymidine kinase cassette; Neo, phosphoglycerate kinase (PGK) promoter-driven neomycin resistance gene; Frt, flippase recognition target sequence; LoxP, Cre-recombinase recognition target sequence; F1 & F2, PCR primer set to evaluate the presence of the downstream LoxP site; M1 & M2, PCR primer set to evaluate the presence of *alb*-Cre transgene; M3 & M4; PCR primer set to evaluate the presence of wild type *alb* allele; N1 & N2, PCR primer set to evaluate the presence of the neomycin resistant gene (Neo); Probes-1 & -2, Northern blot probes; ES, embryonic stem cells; B43*, an ES cell clone harboring successful homologous recombination; B61, another ES cell clone harboring successful homologous recombination; HET, mouse heterozygous for LoxP-fortilin-LoxP allele (fortilin$^{flox/wild}$); HOMO, mouse homozygous for LoxP-fortilin-LoxP allele (fortilin$^{flox/flox}$); WT, mouse lacking any LoxP-fortilin-LoxP allele (fortilin$^{wild/wild}$); PCR, polymerase chain reaction; fortilin$^{WT\text{-}liver}$, mice expressing normal amounts of fortilin in the liver (also WT); fortilin$^{KO\text{-}liver}$, mice lacking fortilin expression in the liver (also KO); FT, fortilin; A.U., arbitrary units; P-JNK, phosphorylated JNK; ALB, albumin; ALP, alkaline phosphatase, AMY, amylase; BUN, blood urea nitrogen; CA^{2+}, calcium; PHOS, phosphorus; CRE, creatinine; GLU, glucose; NA^{+}, sodium; K^{+}, potassium, TP, total protein; GLOB, globulin; TBIL, total bilirubin; TUNEL, terminal deoxynucleotidyl transferase dUTP nick end labeling; Scale bar = 50 µm; Data were expressed as means ± s.d. and analyzed by two-tailed unpaired t-test; n, the number of biological replicates; NS, not statistically significant; *, $P < 0.05$; **, $P < 0.01$; ***, $P < 0.005$. (See also Fig. 4.) (**A**) Generation of fortilin$^{flox/flox}$ mice. Fortilin$^{flox/flox}$ mice—in which the fortilin gene is flanked by the LoxP sequence to allow tissue-specific deletion—were generated using the standard homologous recombination technique. See text for detail. (**B**) Identification of ES cell clones with successful homologous recombination and fortilin$^{flox/flox}$ mouse by Southern blot analysis. Southern blot analyses were performed on NsiI/NdeI digested genomic DNA from ES cell clones or mouse tails using radioactive DNA probes (Probe 1 or Probe 2, see Supplementary Fig. 4A). (**C**) PCR-based genotyping of fortilin$^{KO\text{-}liver}$ mice. A PCR-based-genotyping protocol was established to detect the presence of the Cre transgene under the Alb liver-specific promoter (using primer set M1 and M2) and the presence of the LoxP-fortilin-LoxP knockin allele (using primer set F1 and F2). (**D**) Protection by fortilin against EGF-SubA-induced liver damage as assessed by blood chemistry. Whole blood was sampled by cardiocentesis from PBS or EGF-SubA-challenged fortilin$^{WT\text{-}liver}$ and fortilin$^{KO\text{-}liver}$ mice and subjected to blood chemistry profiling. Three and six biological replicates per each group (WT or KO) per experiment (PBS and EGF-SubA, respectively). The representative data from one independent experiment are shown. (**E**) Inhibition by fortilin of JNK phosphorylation by IRE1α. ELISA was performed on the total lysates from the livers of fortilin$^{WT\text{-}liver}$ and fortilin$^{KO\text{-}liver}$ mice challenged by EGF-SubA. Bar graphs show the protein concentrations of JNK and P-JNK. Four biological replicates per each group (WT or KO) per experiment. The representative data from two independent experiments are shown. (**F**) Protection by fortilin against EGF-SubA-induced liver damage as assessed by hematoxylin and eosin (H&E) staining. H&E staining of the livers of PBS or EGF-SubA-challenged fortilin$^{WT\text{-}liver}$ and fortilin$^{KO\text{-}liver}$ mice was graded by an experienced hepatopathologist in a blinded fashion. The sections from SubA-treated fortilin$^{KO\text{-}liver}$ mice, when compared with those from SubA-treated fortilin$^{WT\text{-}liver}$ mice, showed a pale midzonal pattern of injury characterized by diffuse swelling of the hepatocytes with abundant intracytoplasmic giant mitochondria and focal Mallory's hyaline, correlating with the pale appearance of the liver under gross examination. (**G**) Minimum apoptosis and IRE1α and JNK phosphorylation in the livers of PBS-treated fortilin$^{WT\text{-}liver}$ and fortilin$^{KO\text{-}liver}$ mice. The livers of PBS-challenged fortilin$^{WT\text{-}liver}$ and fortilin$^{KO\text{-}liver}$ mice were subjected to TUNEL and P-IRE1α and P-JNK staining using 3,3'-diaminobenzidine (DAB, brown) as the chromogen. Graphs show the levels of DAB-positive antigen (n = 2 for fortilin$^{WT\text{-}liver}$ mice, n = 3 for fortilin$^{KO\text{-}liver}$ mice).

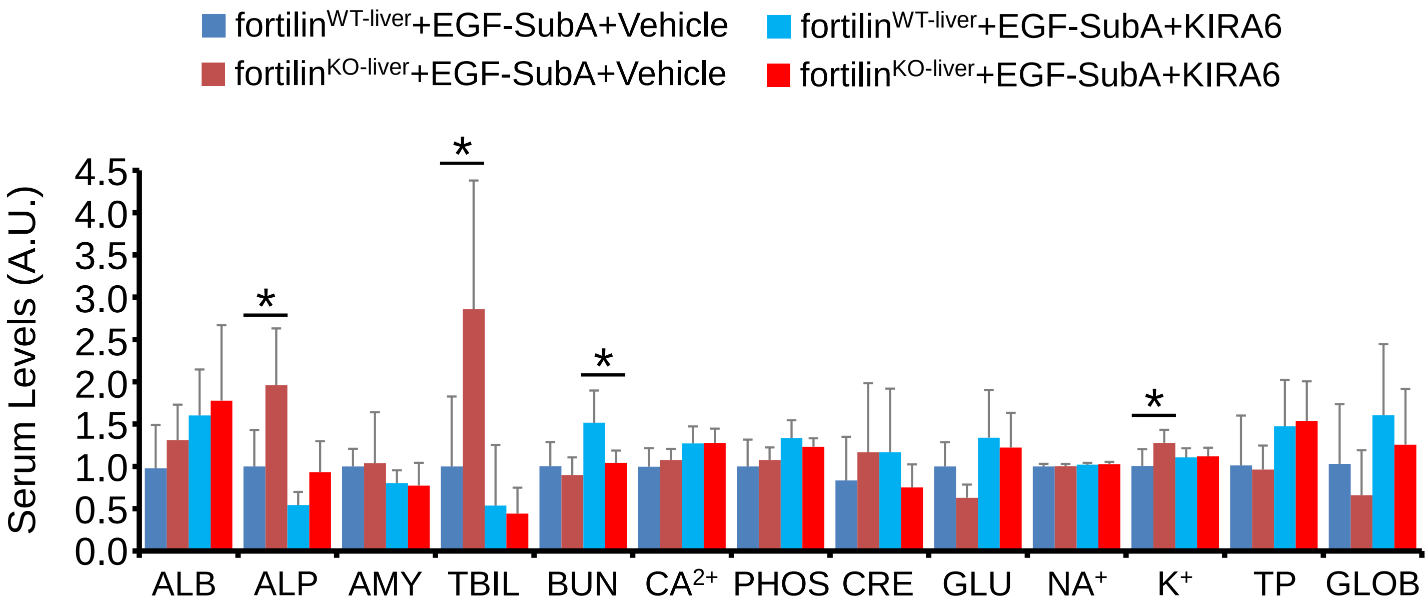

Supplementary Figure 5. **KIRA6 treatment prevents mice lacking fortilin in the liver from developing SubA-induced ER stress-mediated liver injury.** Abbreviations: ALB, albumin; ALP, alkaline phosphatase, AMY, amylase; TBIL, total bilirubin; BUN, blood urea nitrogen; CA^{2+}, calcium; PHOS, phosphorus; CRE, creatinine; GLU, glucose; NA^{+}, sodium; K^{+}, potassium, TP, total protein; GLOB, globulin; KIRA6, IRE1α kinase inhibiting RNAse attenuator-6 (a small-molecular weight inhibitor of IRE1α, CAS# 1589527-60-0); Data were expressed as means ± s.d. and analyzed by two-tailed unpaired t-test; *, $P < 0.05$; six biological replicates per each group per experiment; the representative data from one independent experiment are shown (See also Fig. 5.) The elevation of ALP and TBIL seen in EGF-SubA-challenged mice lacking fortilin in the liver was no longer present when treated by KIRA6.

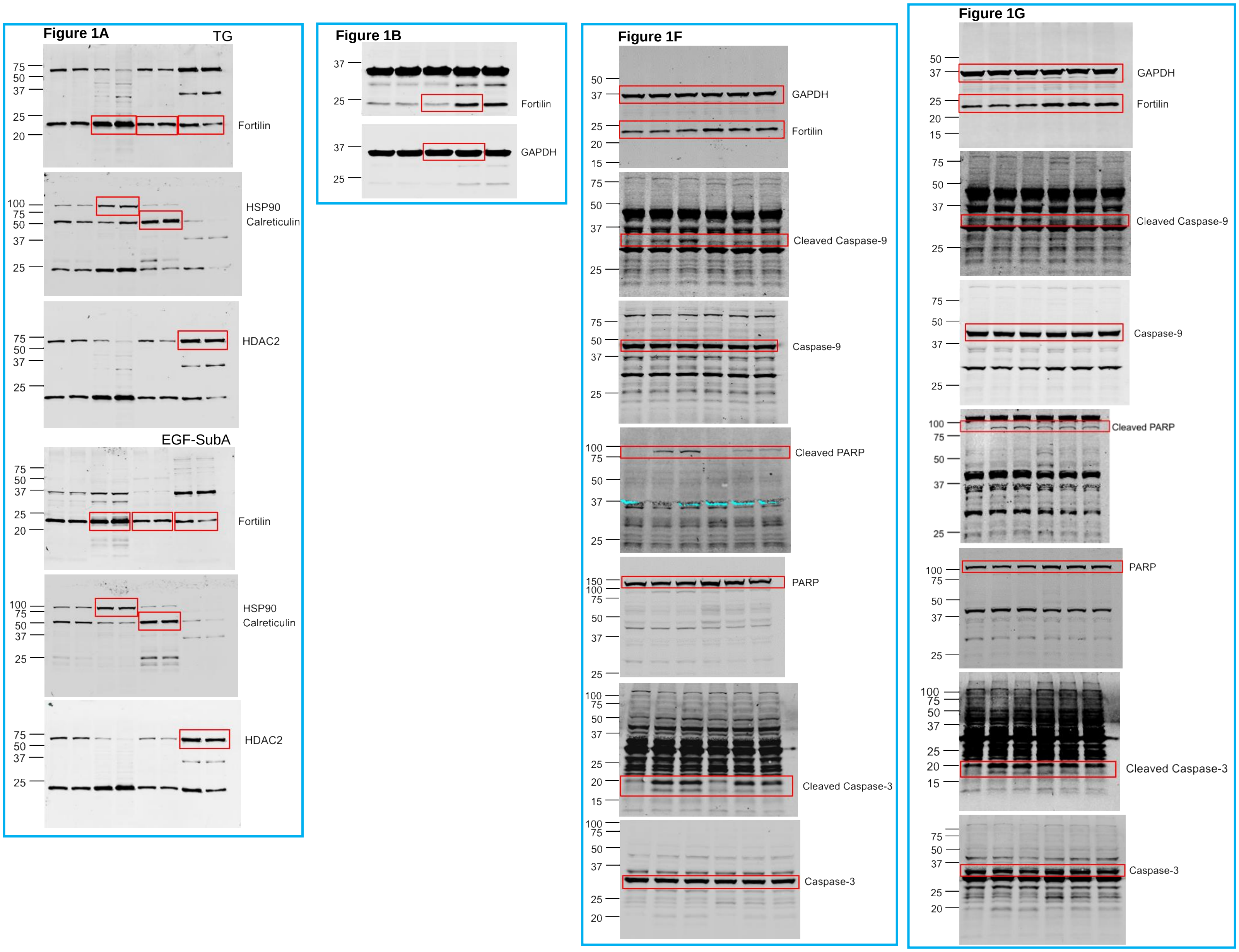

Supplementary Fig. 6. **Uncropped images for immunoblots for Figs. 1A, 1B, 1F, and 1G.** Red boxes show approximate image areas used for final figures.

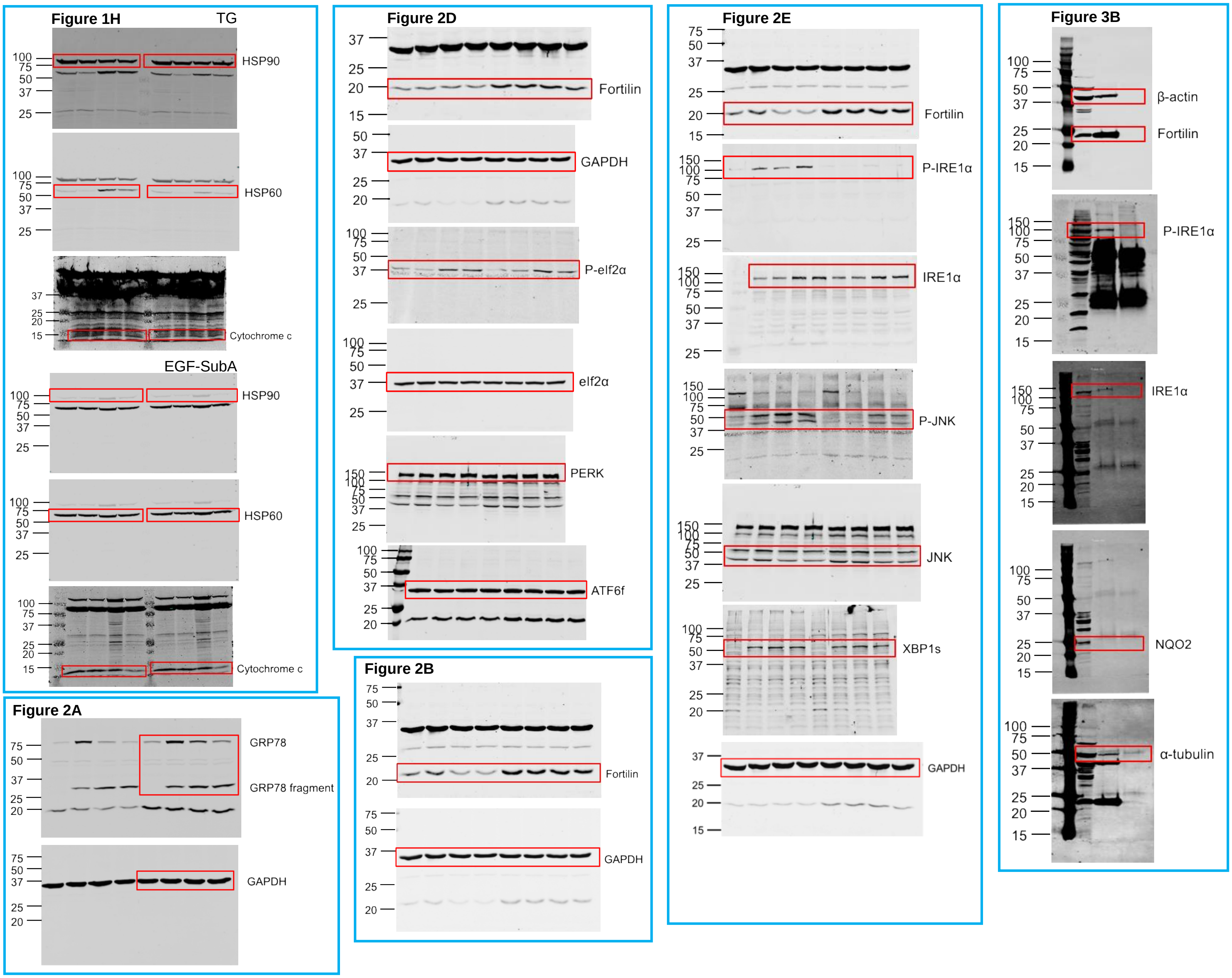

Supplementary Fig. 7. **Uncropped images for immunoblots for Figs. 1H, 2A, 2B, 2D, 2E, and 3B.** Red boxes show approximate image areas used for the final figures.

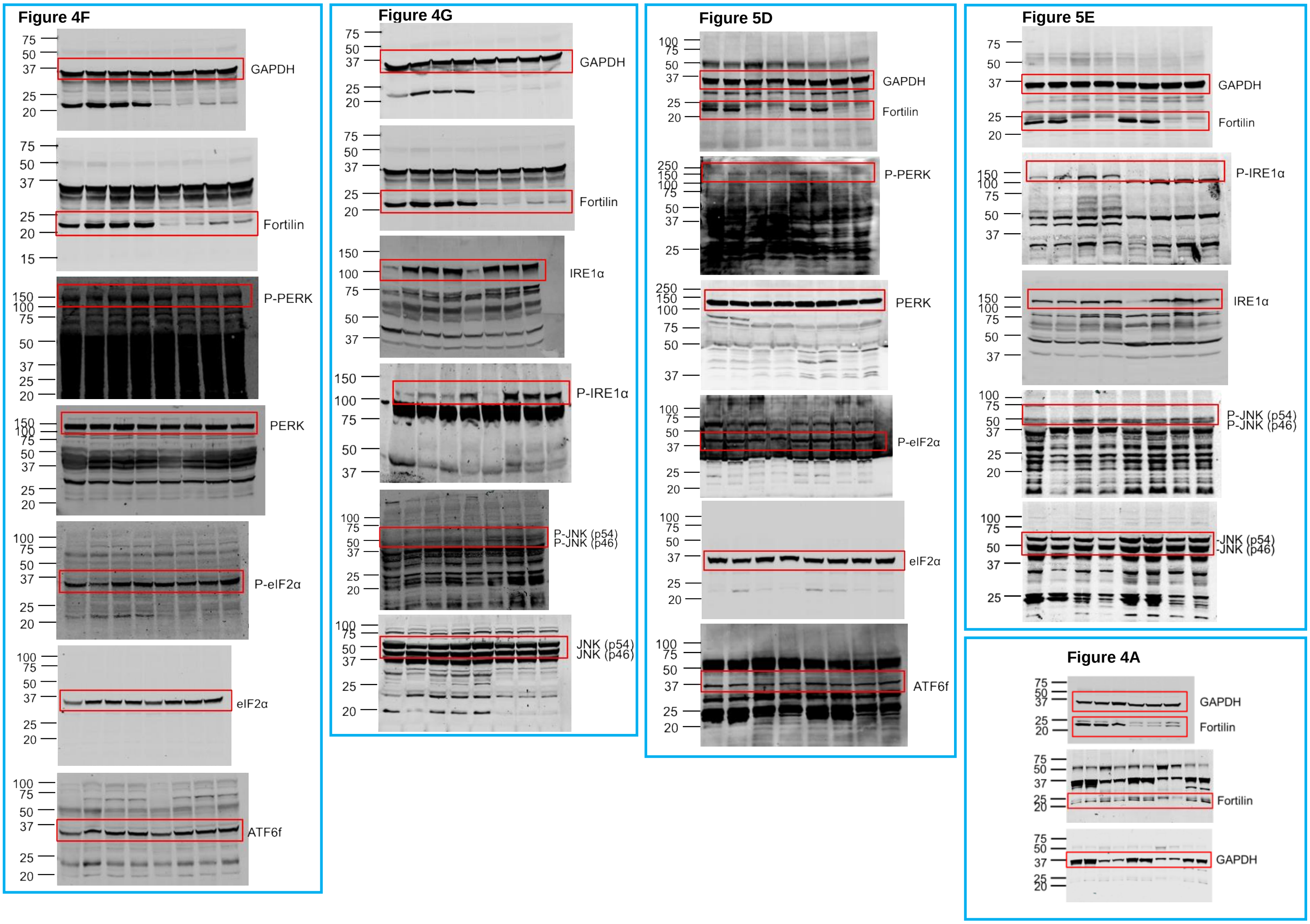

Supplementary Fig. 8. **Uncropped images for immunoblots for Figs. 4A, 4F, 4G, 5D, and 5E.** Red boxes show approximate image areas used for final figures.

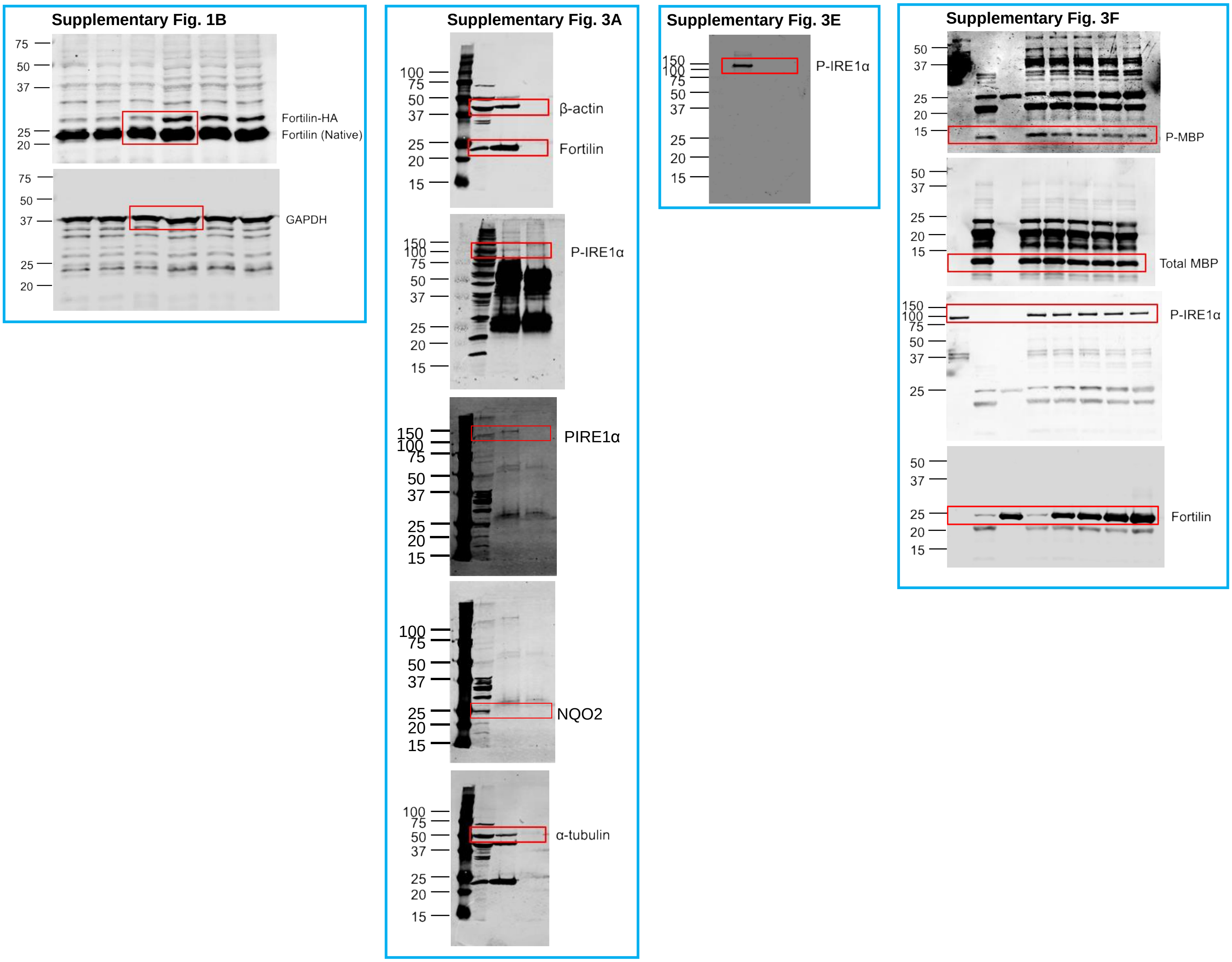

Supplementary Fig. 9. **Uncropped images for immunoblots for Supplementary Figs. 1B, 3A, 3E and 3F.** Red boxes show approximate image areas used for final figures.

Supplementary Table 1

Changes in Free Energy of S724 and S726 Phosphorylation of IRE1α in the Bound State with Fortilin and Unbound in Solution Estimated by Molecular Dynamics Forward and Reverse Binding Free Energy Simulations.

	**Forward**	**Reverse**	**Average**
Fortilin:4U6R	–601.91 (1.17)[a]	603.30 (0.83)	–602.61 (1.02)
4U6R	–587.57 (1.07)	586.68 (0.94)	–587.13 (1.01)

[a]kcal/mol; s.e. in parenthesis

Supplementary Table 2

Complete Blood Counts of Mice Treated by PBS or EGF-SubA

Parameter	PBS			EGF-SubA		
	WT (n = 2)	KO (n = 4)	*P*-value	WT (n = 5)	KO (n = 9)	*P*-value
White blood cell (K/μL)	1.7 ± 0.9	3.6 ± 1.5	0.195	5.6 ± 1.6	7.0 ± 2.4	0.265
Neutrophil (%)	13.3 ± 3.2	15.2 ± 6.8	0.729	20.0 ± 10.1	31.6 ± 8.9	0.046*
Lymphocyte (%)	85.5 ± 4.0	79.7 ± 6.3	0.487	72.7 ± 13.8	49.1 ± 9.3	0.002***
Monocyte (%)	2.1 ± 0.1	4.0 ± 0.7	0.025*	4.2 ± 2.0	4.7 ± 2.4	0.662
Eosinophil (%)	0.9 ± 0.9	0.9 ± 0.8	0.978	2.5 ± 2.0	12.2 ± 4.7	0.001***
Basophil (%)	0.2 ± 0.1	0.2 ± 0.2	0.929	0.7 ± 0.5	2.3 ± 1.2	0.013*
Red blood cell (M/μL)	9.4 ± 0.3	9.5 ± 0.5	0.805	9.6 ± 1.4	9.4 ± 1.5	0.831
Hemoglobin (g/dL)	12.3 ± 0.4	12.6 ± 1.1	0.722	12.0 ± 1.9	12.2 ± 2.0	0.851
Hematocrit (%)	44.8 ± 0.6	47.6 ± 4.4	0.440	44.8 ± 7.2	45.8 ± 7.1	0.819
Mean corpuscular volume (fL)	47.6 ± 2.2	49.9 ± 2.1	0.277	46.9 ± 1.4	48.9 ± 1.1	0.011*
Mean corpuscular hemoglobin (pg)	13.0 ± 0.0	13.2 ± 0.6	0.738	12.5 ± 0.2	13.0 ± 0.5	0.044*
Mean corpuscular hem. conc. (K/μL)	27.4 ± 1.2	26.4 ± 0.5	0.210	26.8 ± 0.6	26.6 ± 0.8	0.658
Red blood cell distribution width (K/μL)	17.5 ± 0.2	18.2 ± 0.7	0.260	18.1 ± 0.7	16.8 ± 0.6	0.002***
Platelet (K/μL)	481.0 ± 79.2	188.5 ± 114.0	0.034*	505.2 ± 287.3	196.8 ± 154.6	0.021*
Mean platelet volume (fL)	4.6 ± 0.0	5.9±1.1	0.167	5.3 ± 0.7	5.4 ± 0.5	0.598

Data are expressed as means ± s.d. (n = biological replicates) and analyzed by two-tailed unpaired t-test. *, $P < 0.05$; **, $P < 0.01$; ***, $P < 0.005$. Wild-type and knockout mice were injected with PBS (n = 2, n = 4, respectively) or EFG-SubA (0.25 μg/g BW; n = 5, n = 9, respectively). Blood was collected and analyzed for complete blood count using HEMAVET 950FS Hematology System (Drew Scientific, Dallas, TX).

Supplementary Table 3

Complete Blood Counts of EGF-SubA-treated Mice with and without KIRA6

Parameter	EGF-SubA-Vehicle			EGF-SubA-KIRA6		
	WT (n = 6)	KO (n = 5)	*P*-value	WT (n = 6)	KO (n = 6)	*P*-value
White blood cell (K/μL)	4.7 ± 1.3	4.8 ± 2.7	0.923	7.6 ± 5.3	8.4 ± 4.7	0.780
Neutrophil (%)	34.2 ± 3.4	47.3 ± 12.6	0.033*	37.6 ± 7.6	42.6 ± 7.3	0.296
Lymphocyte (%)	47.9 ± 4.5	44.7 ± 8.9	0.441	51.6 ± 13.8	43.3 ± 8.5	0.252
Monocyte (%)	5.4 ± 2.0	2.8 ± 1.8	0.042*	3.5 ± 1.1	4.3 ± 1.8	0.384
Eosinophil (%)	10.4 ± 3.2	4.2 ± 2.4	0.004***	5.8 ± 6.4	7.4 ± 5.2	0.662
Basophil (%)	2.1 ± 0.7	1.0 ± 0.6	0.013*	1.5 ± 1.8	2.5 ± 2.4	0.502
Red blood cell (M/μL)	7.2 ± 1.4	10.2 ± 1.5	0.005**	8.0 ± 2.1	8.6 ± 1.5	0.592
Hemoglobin (g/dL)	11.4 ± 3.0	16.5 ± 2.2	0.007**	13.3 ± 3.1	14.0 ± 2.8	0.740
Hematocrit (%)	35.3 ± 6.9	51.2 ± 9.1	0.007**	40.7 ± 11.0	43.5 ± 8.2	0.634
Mean corpuscular volume (fL)	49.4 ± 2.3	50.0 ± 2.5	0.688	50.8 ± 4.3	50.4 ± 1.9	0.836
Mean corpuscular hemoglobin (pg)	15.7 ± 1.3	16.2 ± 0.4	0.464	16.8 ± 1.1	16.1 ± 0.9	0.314
Mean corpuscular hemo. conc. (K/μL)	31.9 ± 3.3	32.4 ± 2.1	0.758	33.3 ± 4.4	32.0 ± 2.6	0.568
Red blood cell distribution width (K/μL)	16.5 ± 1.1	18.1 ± 0.6	0.008**	19.6 ± 2.5	18.0 ± 1.1	0.171
Platelet (K/μL)	361.0 ± 141.7	621.7 ± 114.9	0.006**	599.0 ± 243.3	452.7 ± 245.2	0.349
Mean platelet volume (fL)	5.3 ± 0.3	4.9 ± 0.4	0.098	5.3 ± 0.3	5.3 ± 0.7	0.920

Data are expressed as means ± s.d. (n = biological replicates) and analyzed by two-tailed unpaired t-test. *, *P* < 0.05; **, *P* < 0.01; ***, *P* < 0.005. Wild-type and knockout mice were injected with Vehicle (n = 5 for WT; n = 6 for KO) or KIRA6 (5 mg/kg BW, n = 6) for 9 days. Mice were challenged with EFG-SubA (0.25 μg/g BW; n = 6) at day 3. Blood was collected and analyzed for complete blood count using HEMAVET 950FS Hematology System (Drew Scientific, Dallas, TX).

Supplementary Table 4

Values for Coulomb and van der Waals λ Used for Intermediate States in Free Energy Calculations

Coulomb	van der Waals
0.02545	0.00344
0.12923	0.01801
0.29708	0.04388
0.50000	0.08044
0.70292	0.12683
0.87077	0.18197
0.97455	0.24457
	0.31315
	0.38611
	0.46174
	0.53826
	0.61389
	0.68685
	0.75543
	0.81803
	0.87317
	0.91956
	0.95612
	0.98199
	0.99656
